# Industrial-scale fractionation of fava bean, chickpea, and red lentil: A comparative analysis of composition, antinutrients, nutrition, structure, and functionality

**DOI:** 10.1016/j.crfs.2025.101152

**Published:** 2025-07-22

**Authors:** Ruixian Han, Yan Wang, Zhanming Yang, Stuart Micklethwaite, Martin Mondor, Evi Paximada, Alan Javier Hernández-Álvarez

**Affiliations:** a School of Food Science and Nutrition, University of Leeds, Leeds, LS2 9JT, UK; b School of Chemical and Process Engineering, University of Leeds, LS2 9JT, UK; c Department of Chemical Engineering and Biotechnological Engineering, Université de Sherbrooke, Sherbrooke, QC, J1K 2R1, Canada; d Institute of Nutrition and Functional Foods (INAF), Université Laval, Quebec, QC, G1V 0A6, Canada; e National Alternative Protein Innovation Centre (NAPIC), UK

**Keywords:** Legumes, dry fractionation, wet fractionation, antinutritional factors, protein profile and structure, protein quality, techno-functional properties

## Abstract

Legumes are emerging as sustainable protein sources that can replace animal proteins and help meet global dietary needs. This study systemically compared the compositional profiles, antinutritional factors, amino acid profiles, protein quality, structural characteristics, and techno-functional properties of fava bean, chickpea, and red lentil flours, along with their dry- and wet-fractionated protein-enriched fractions (PFs). Wet-fractionated PFs exhibited higher protein content (58.36 – 83.79 g/100 g), while dry-fractionated PFs retained more total dietary fibre (7.62 – 14.64 g/100 g). Wet-fractionated fava bean (84.12 %) and red lentil (84.06 %) showed the highest *in vitro* protein digestibility (IVPD), while dry-fractionated chickpea showed the highest IVPDCAAS at 62.43 %. The protein composition was generally preserved after fractionation, though changes in secondary structure varied depending on legume source. Surface hydrophobicity (H_0_ 62,739 – 99,381) increased following wet fractionation. In terms of functionality, wet-fractionated PFs showed the highest water-holding capacity (2.83 g/g, red lentil), foaming capacity (139.1 %, fava bean) and emulsifying capacity (108.1 m^2^/g, red lentil), but with relatively poor foaming and emulsifying stability. Conversely, dry-fractionated PFs exhibited higher protein solubility, lower least gelation concentration (8–10 %), and superior oil-holding capacity (3.98 g/g, Chickpea), likely due to reduced structural disruption, which limited protein aggregation and denaturation. Despite higher levels of antinutritional factors, dry fractionation emerges as a promising, cost-effective, and sustainable technology to produce legume protein concentrates with improved functionality and nutritional quality comparable to those obtained by wet-fractionated.

## Highlights

• Fractionation was effective to increase protein content in fava bean and red lentil.
• Antinutrients increased post-fractionation but did not affect protein digestibility.
• Dry fractionation (DF) preserved protein structure and improved functionality.
• Wet fractionation (WF) disrupted protein and reduced foaming/emulsifying stability.
• DF significantly boosts nutritional value of chickpea, raising IVPDCAAS by 40.1 %.

## Graphical abstract

**Figure:**
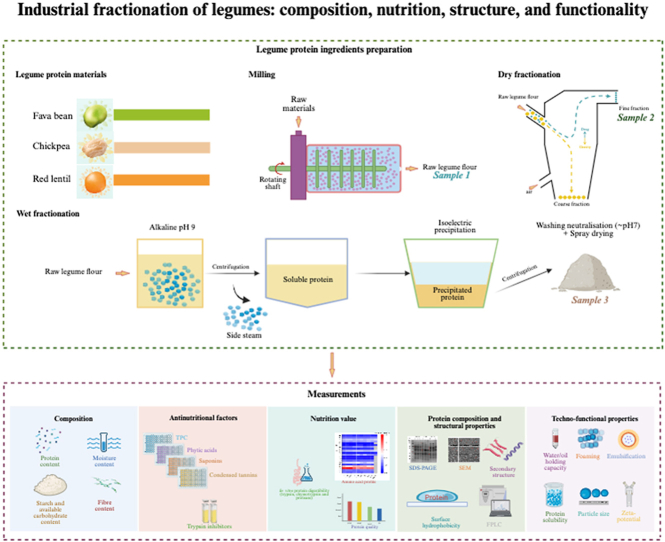

## 1 Introduction

Among seed plants, legumes rank as the second-largest group and are important contributors of plant-based proteins (Duranti, 2006). Soybean is the major legume crop produced worldwide, while peanuts, cowpeas, fava beans, lupins, chickpeas, and lentils are also significant legume crops (Semba et al., 2021). Legumes are rich in protein, with a crude protein content ranging from 17 % to 30 % (Goldstein and Reifen, 2022), presenting higher protein content and improved protein digestibility compared to cereals (Xu et al., 2023). Legumes provide most essential amino acids, with particularly high level of lysine, but they are generally deficient in sulphur-containing amino acids (methionine and cysteine) and tryptophan (Iqbal et al., 2006; Sánchez-Velázquez et al., 2021). Legumes are also abundant in fibres, vitamins (e.g., B vitamins) and minerals (e.g., iron, magnesium, potassium, and zinc) (Erbersdobler et al., 2017). Although the potential health benefits of bioactive compounds, such as phytates and trypsin inhibitors, have been documented, these compounds hinder the absorption of essential minerals and proteins, thereby negatively affecting the overall nutritional quality of legumes (Manzanilla-Valdez et al., 2024c). Despite this, considering nutritional benefits, low cost, sustainability, low allergenicity, and good consumer acceptability, legume proteins present an unlimited potential for their incorporation into a wide range of food products.

Considering the large amount of carbohydrates (ranging from 40 % to 64 %) and other non-proteinaceous components in legumes (Affrifah et al., 2023), fractionation methods were developed to increase the protein content and modify functional properties and nutritional qualities. Wet fractionation is the conventional route for producing protein concentrates or isolates from raw flours, with protein contents that can exceed 90 % (Boye et al., 2010b; Mondor and Hernández-Álvarez, 2022). This processing involves the following steps: 1) hydrating flour in water to obtain protein suspension; 2) adjusting the pH to an alkaline condition (e.g., pH 9 – 10) to extract the proteins; 3) centrifugation to remove insoluble fibres and other insoluble components; 4) adjusting the pH to the isoelectric point of the proteins (pH around 4.5) to allow their precipitation; 5) centrifugation to recover the precipitated proteins; and 6) neutralizing the pH of protein solution to 7, followed by spray drying or freeze drying. The length of protein enrichment, excessive water usage and high energy requirements has raised concerns regarding sustainability and the need of novel green extraction methods (Assatory et al., 2019). Meanwhile, harsh conditions (pH and spray drying temperature) lead to protein aggregation and denaturation, consequently resulting in the loss of functionalities (Ho et al., 2021).

On the contrary, dry fractionation requires less energy, no additional water, and avoids chemical exposure. This process involves milling and air classification. During milling, starch granules liberated from the flour are larger than the fragmented protein matrix. These granules and fragments are subsequently separated by air classification based on differences in size and density. Air currents are continuously fed into the classifier chamber, where centrifugal force and gravity effectively separate fine fractions (smaller, protein-rich fraction) from coarse particles (larger, starch-rich fraction) (Pulivarthi et al., 2023). Compared to wet fractionation, which can yield protein content up to 90 %, the protein purity of ingredients obtained through dry fractionation is relatively low, typically ranging between 40 % and 60 % (Dumoulin et al., 2021). Commercial food products rarely contain high protein content, which means protein-enriched ingredients with moderate protein concentrations are acceptable for product development (Tabtabaei et al., 2016). This explains the increasing interest in applying dry fractionation for producing protein-enriched ingredients for food application.

Both dry and wet fractionation have been widely investigated, focusing on protein content, techno-functional properties, protein profiles (e.g., SDS-PAGE), structure (e.g., scanning electron microscopy (SEM)) and amino acid composition (Dumoulin et al., 2021, Pelgrom et al., 2013, Schlangen et al., 2022) Some studies have also examined the content of antinutritional factors and suggested the potential of these compounds to reduce protein digestibility (Amin et al., 2022, Schutyser et al., 2025). However, the impact on protein digestibility and protein quality has rarely been validated. In addition, a recent published comprehensive study examined eight wet-fractionated and nine dry-fractionated protein ingredients, performing multiple measurements to characterize and compare their properties (De Angelis et al., 2024). Although, it is important to note that these protein ingredients were sourced from different commercial suppliers, which may have introduced significant variability in their physicochemical properties due to differences in extraction and production processes. Therefore, a standardized and systematic comparison of the effects of dry and wet fractionation methods on protein ingredients is urgently needed. In this study, the impact of dry and wet fractionation on the nutritional, structural, and techno-functional properties of fava bean, chickpea, and red lentil were systematically investigated and compared. Specifically, this research aimed at evaluating the impact of both processing methods on: 1) protein content, starch content, available carbohydrate, and total dietary fibre; 2) antinutritional factors, including total phenolic content, phytic acid, condensed tannins, saponins, and trypsin inhibitors; 3) the protein quality of processed ingredients, with a comprehensive evaluation of amino acid profiles, *in vitro* protein digestibility (IVPD), amino acid score (AAS), essential amino acid index (EAAI), biological value (BV), protein efficiency ratio (PER), and *in vitro* protein digestibility-corrected amino acid score (IVPDCAAS); 4) structural-related properties, for instance, particle size, zeta-potential, microstructure, secondary structure, and surface hydrophobicity; 5) techno-functional properties, such as water holding capacity, oil holding capacity, foam properties, emulsifying properties, protein solubility, and gelation. The effects of dry and wet fractionation on all measured parameters of legume flours were assessed using Pearson correlation analysis. Additionally, principal component analysis (PCA) was employed to evaluate the characteristics of legume flours before and after dry and wet fractionation.

## 2 Materials and methods

### 2.1 Materials

DL-dithiothreitol (DTT), methanol, hydrochloric acid, sulfuric acid and acetic acid glacial were purchased from Fisher Chemical (Loughborough, United Kingdom). Formic acid, ethylenediaminetetraacetic acid (EDTA), sodium phosphate monobasic monohydrate, sodium phosphate dibasic, Folin & Ciocalteu's phenol reagent, sodium carbonate, gallic acid, Iron (III) chloride hexahydrate, 5-sulfosalicylic acid hydrate, citric acid, Nα-benzoyl-L-arginine 4-nitroanilide hydrochloride (BAPNA), 8-anilino-1-naphthalenesulfonic acid ammonium salt (ANS), dimethyl sulfoxide (DMSO), trypsin from porcine pancreas (13,000 – 20,000 BAEE units/mg protein), chymotrypsin from bovine pancreas (≥ 40 units/mg protein), protease from Streptomyces griseus (≥ 3.5 units/mg solid) were purchased from Sigma-Aldrich (Gillingham, United Kingdom). Sodium hydroxide and calcium chloride dihydrate were purchased from VWR chemicals (Lutterworth, United Kingdom). Sodium chloride was purchased from Avantor Sciences (Lutterworth, United Kingdom). Sodium phytate and ammonium sulfate were purchased from ChemCruz® biochemicals (TE Huissen, the Netherlands). Diosgenin was purchased from Fluorochem (Hadfield, United Kingdom). Vanillin (BS-6341P) was purchased from BioServTM (Rotherham, United Kingdom). Catechin was purchased from Merck (Gillingham, United Kingdom).

### 2.2 Sample collection

Raw flours of commercial fava bean, chickpea, and red lentil, as well as their protein-enriched fractions obtained through dry and wet fractionation, respectively, were gifted by Deltagen UK (Highbridge Somerset, UK). All powders were finely ground. An overview of colour measurement of all ingredients is provided in Appendix A.1.

### 2.3 Protein content, total starch content, available carbohydrate, and total dietary fibre content

The protein content of protein ingredients was determined according to AOAC (1990), using a conversion factor of 6.25 to convert nitrogen level to protein content. The moisture content was determined by drying 2 g of protein ingredients at 110 °C in a DRY-Line Oven (VWR) until a constant weight was achieved (Chasquibol et al., 2025). The total starch content of protein ingredients was measured using Total Starch Assay Kit (AA/AMG) (Megazyme, K-TSTA-100A). The available carbohydrate and total dietary fibre were quantified using Available Carbohydrates/Dietary Fiber Assay Kit (Megazyme, K-ACHDF).

### 2.4 Total phenolic content and antinutritional factors

#### 2.4.1 Total phenolic content

Total phenolic content (TPC) in protein ingredients was determined using Folin-Ciocalteu assay, according to Pico et al. (2020), with some modifications. One gram of protein ingredient was extracted with 12 mL of 80 % methanol in 0.1 % formic acid, followed by sequential extraction with same volume of 70 % acetone in 0.1 % formic acid. Ten microliters of combined extract solutions were mixed with 40 μL of Folin reagent (25 % Folin-Ciocalteu reagent in water), and 150 μL of 4 % sodium carbonate. After incubation for 30 min at room temperature in the dark, the absorbance was measured at 765 nm. Gallic acid was used as standard, with concentrations ranging from 15.625 to 500 μg/mL.

#### 2.4.2 Phytic acid

Phytic acid in protein ingredients was determined according to Manzanilla-Valdez et al. (2024b). Half a gram of protein ingredient was extracted with 10 mL of 2.4 % HCL using a platform shaker (Heidolph Orbital) for 16 h at 220 rpm. The supernatant was collected after centrifugation at 5,000 rpm (ROTINA 380, Hettich) at 4 °C for 20 min, then mixed with 1 g of NaCl. The mixture was shaken for 20 min at 350 rpm (Orbital Shaker 36508, Heidolph) and then placed at 4 °C for 1 h. After 25-fold dilution using Milli-Q water, 150 μL of the extracted solution was mixed with 50 μL of Wade reagent (0.03 g of ferric chloride hexahydrate and 0.3 g of sulfosalicylic acid in 100 mL of Milli-Q water). Following a 10 min reaction time, the absorbance was measured at 500 nm. Sodium phytate was used as standard (ranging from 0.0375 to 0.6 mg/mL), with a phosphorus content of 18.38 % (Hande et al., 2013).

#### 2.4.3 Condensed tannins

Condensed tannins in protein ingredients were measured according to De Mejia et al. (2005), with some modifications. One gram of protein ingredient was extracted with 10 mL of 4 % HCL in methanol using a platform shaker (Heidolph Orbital) for 18 h at 400 rpm. After centrifugation at 5,000 rpm (ROTINA 380, Hettich) for 10 min at 4 °C, 50 μL of supernatant was mixed with 100 μL of 10 % sulfuric acid in methanol. Then, 100 μL of 1 % vanillin in methanol was added to the mixture, which was incubated at room temperature for 15 min before absorbance measurement at 500 nm. Catechin was used as the standard, with concentrations ranging from 0.25 to 1 mg/mL.

#### 2.4.4 Saponins

Saponins in protein ingredients were determined according to Manzanilla-Valdez et al. (2024b), with some modifications. Half a gram of protein ingredients was extracted with 10 mL of 80 % methanol using a platform shaker (Orbital Shaker 36508, Heidolph) for 16 h at 400 rpm. The supernatant was collected after centrifugation at 5,000 rpm (ROTINA 380, Hettich) for 10 min. The pellet was washed twice by 5 mL of 80 % methanol, and the wash solution was combined with the supernatant for saponin determination. Two-hundred microliters of saponin extract solution was mixed with 50 μL of 80 % methanol, 0.25 mL of vanillin reagent (1.6 g of vanillin dissolved in 20 mL of absolute methanol), and 2.5 mL of 72 % sulfuric acid. The mixture was heated in a water bath at 60 °C for 10 min, the absorbance was determined at 520 nm. Diosgenin was used as standard, with concentrations ranging from 0.1 to 0.5 mg/mL.

#### 2.4.5 Trypsin inhibitors

Trypsin inhibitor activities (TIA) in protein ingredients were analysed according to Liu et al. (2021), with some modifications. Half a gram of protein ingredients was extracted with 25 mL of 10 mM NaOH for 3 h at 400 rpm (Orbital Shaker 36508, Heidolph) at room temperature. After centrifugation at 5000 rpm for 10 min (ROTINA 380, Hettich), the supernatant was collected and diluted using Milli-Q water, exhibiting trypsin inhibition ranging from 30 to 70 %. One millilitre of diluted supernatant was mixed with 2.5 mL of benzyl-DL-arginine-para-nitroanilide (BANPA) solution (200 mg of BANPA dissolved in 5 mL of DMSO, then diluted 100-fold using 50 mM Tris buffer containing 20 mM CaCl_2_, pH 8.2). After adding 1 mL of trypsin solution (1 mg of trypsin in 50 mL of 1 mM HCL solution containing 5 mM CaCl_2_) and incubated for 10 min at 37 °C, the reaction was terminated using 0.5 mL of acetic acid solution (30 % v/v). The absorbance was measured at 410 nm after centrifugation at 3500 × *g* for 5 min at room temperature. The reference was prepared by replacing the diluted supernatant with an equal volume (1 mL) of Milli-Q water. Acetic acid added prior to the addition of trypsin solution to sample and reference solution were considered as sample blank and reference blank, respectively. TIA was calculated using the following equation:

TIA (TIU/mg)= $\frac{\left\{\left[\left({Abs}_{reference}-{Abs}_{reference\;blank}\right)-\left({Abs}_{sample}-{Abs}_{sample\;blank}\right)\right]\times 50\right\}}{1mL\times concentration\;of\;samples\;in\;diluted\;extract\;\left(mg/mL\right)}$

### 2.5 *In vitro* protein digestibility

*In vitro* protein digestibility (IVPD) of protein ingredients were determined according to Wang et al. (2023a). A protein ingredient containing 62.5 ± 0.5 mg of protein was dissolved in 10 mL of Milli-Q water, and the pH was adjusted to 8.0 at 37 °C. Meanwhile, a 10 mL of multienzyme cocktail with 31 mg of chymotrypsin (P40 Units/mg protein), 16 mg of trypsin (13,000 – 20,000 BAEE units/mg protein) and 13 mg of protease from Streptomyces griseus (P3.5 units/mg) were prepared, and the pH was adjusted to 8.0 at 37 °C. After adding 1 mL of multienzyme cocktail (pH 8.0) to the protein ingredient solution, the pH was recorded for 10 min. The IVPD of protein ingredients was calculated as follows:

IVPD (%) = 65.66 + 18.10 **×** (pH_0 min_ – pH_10 min_)

### 2.6 Amino acid profiles and protein quality

Amino acid profiles of protein ingredients were determined using HPLC with a 300 mm **×** 3.9 mm. i.d. reversed-phase C18 column. Two milligrams of protein ingredients were hydrolysed using 6 M HCL at 110 °C for 24 h, followed by derivatisation with diethyl ethoxymethylenemalonate. Specifically, tryptophan was quantified after basic hydrolysis. D,L-α-aminobutyric acid was used as an internal standard (Yust et al., 2004).

Amino acid score (AAS), essential amino acid index (EAAI), predicted biological value (BV), and protein efficiency ratio (PER) was calculated according to the following equations (Sánchez-Velázquez et al., 2021):

AAS = $\frac{mg\;of\;limited\;amino\;acid\;in\;1\;g\;of\;total\;protein}{mg\;of\;this\;amino\;acids\;in\;1\;g\;of\;requirement\;pattern}$

EAAI = $\sqrt[{9}]{\frac{\left[Lys\times Thr\times Val\times \left(Met+Cys\right)\times Ile\times Leu\times \left(Phe+Tyr\right)\times His\times Trp\right]\left(sample\right)}{\left[Lys\times Thr\times Val\times \left(Met+Cys\right)\times Ile\times Leu\times \left(Phe+Tyr\right)\times His\times Trp\right]\left(standard\right)}}$

BV = 1.09 (EAAI) – 11.7

PER_1_ = - 0.684 + 0.456 (Leu) – 0.047 (Pro)

PER_2_ = - 0.468 + 0.454 (Leu) – 0.105 (Tyr)

PER_3_ = - 1.816 + 0.435 (Met) + 0.780 (Leu) + 0.211 (His) – 0.944 (Tyr)

PER_4_ = 0.08084 (Thr + Val + Met + Ile + Leu + Phe + Lys) – 0.1094

PER_5_ = 0.0632 (Thr + Val + Met + Ile + Leu + Phe + Lys + His + Arg + Tyr) – 0.1539

The *in vitro* protein-digestibility corrected amino acid score (IVPDCAAS) was calculated by AAS **×** IVPD (Ma et al., 2024).

### 2.7 Sodium dodecyl sulfate-polycrylamide gel electrophoresis (SDS-PAGE)

The molecular weight distribution of protein ingredients was analysed using SDS-PAGE according to Laemmli (1970), with some modifications. Protein ingredients containing 20 μg of protein was dissolved in 1 **×** Laemmli buffer containing Dithiothreitol (DTT, 15.42 mg/mL). The samples were heated at 95 °C for 5 min, and then centrifuged at 10,000 **×** *g* for 10 min at 4 °C. The supernatant was loaded onto a Criterion TGX Precast gel (Bio-Rad). Electrophoresis was performed at 200 V for 30 min. The gel was washed three times using Milli-Q water, stained with Bio-Safe™ Coomassie stain (Bio-Rad), and analysed using a gel imager system (Gel Doc XR + system, Bio-Rad). Precision Plus Protein™ (10 – 250 kDa, Bio-Rad) was used as a molecular marker.

### 2.8 Scanning electron microscopy (SEM)

The microstructures of protein ingredients were analysed using cold field emission scanning electron microscopy (CFE-SEM, Hitachi SU8230) with a backscattered electron detector, at a magnification 500×. Protein ingredients were attached to a sample holder (named cryo-shuttle), and were then coated with Iridium to a thickness of 15 nm to facilitate good electrical conductivity. Subsequently, the coated samples were transferred into the SEM chamber under high vacuum conditions (>10 e^−7^ mbar). The analysis was performed at a working distance of 15.5 – 16.2 mm and using accelerating voltage of 2 kV.

### 2.9 Protein secondary structure

Secondary structure of protein ingredients was measured using Fourier Transform Infrared Spectroscopy coupled to Attenuated Total Reflectance (FTIR-ATR). Amide I region (1,700 to 1,600 cm^−1^) of dried protein ingredient powder was measured and analysed using peak analysis functionality in OriginPro (2021) (OriginLab Corporation, Northampton, MA, USA).

### 2.10 Surface hydrophobicity

Surface hydrophobicity of protein ingredients was measured according to Manzanilla-Valdez et al. (2024a). Protein ingredients were dissolved in 0.01 M PBS buffer, and the soluble protein concentration in solution was adjusted to 0.01, 0.02, 0.04, 0.06, 0.08 and 0.1 mg/mL (quantified using Pierce™ BCA protein assay kit). One mL of protein solution was mixed with 5 μL of 8 mM 8-anilino-1-naphtalenesulfonic acid ammonium salt solution (ANS) in the dark. The fluorescence intensity of sample with ANS was measured at an excitation wavelength of 360 nm and an emission wavelength of 460 nm. Protein solution without ANS was used as the blank. After subtracting the blank, the linear slope of curve (fluorescence intensity against soluble protein content) was considered as surface hydrophobicity.

### 2.11 Fast protein liquid chromatography (FPLC)

According to Manzanilla-Valdez et al. (2024b), 500 μL of protein ingredients solution containing 0.1 mg protein was injected for gel filtration chromatography, which was carried out using a AKTA-purifer FPLC system equipped with a Superdex peptide 10/300 GL column (Cat: 17-5176-01, GE Healthcare). A 0.75 M ammonium bicarbonate solution was used as eluent, and elution was monitored at 215 nm. Molecular weight standards used were blue dextran (2,000 kDa), cytochrome C (12.5 kDa), aprotinin (6,512 Da), bacitracin (1,450 Da), cytidine (246 Da) and glycine (75 Da).

### 2.12 Techno-functional properties

#### 2.12.1 Water/oil holding capacity

Water holding capacity (WHC) and oil holding capacity (OHC) of protein ingredients were determined using the method described by Boye et al. (2010a), with some modifications. Briefly, for WHC, 1 g of protein ingredient was mixed with 10 mL of Milli-Q water. After shaking for 0.5 h at 300 rpm (Orbital Shaker 36508, Heidolph), the supernatant was removed following centrifugation at 2,000 rpm (ROTINA 380, Hettich) for 30 min. Regarding OHC, half gram of protein ingredient was mixed with 5 mL of soybean oil. After shaking for 0.5 h at 500 rpm (Orbital Shaker 36508, Heidolph), the supernatant was removed following centrifugation at 4,000 rpm (ROTINA 380, Hettich) for 1 h. WHC (g/g) and OHC (g/g) were calculated using the following equation:

WHC (g/g) or OHC (g/g) = $\frac{Weight\;of\;tube\;and\;pellet\;\left(g\right)-weight\;of\;tube\;\left(g\right)-weight\;of\;ingredient\;\left(g\right)}{weight\;of\;ingredient\;\left(g\right)}$

#### 2.12.2 Foaming capacity and stability

Foaming capacity (FC) and stability (FS) of protein ingredients were measured according to Stone et al. (2015), with some modifications. Three-hundred milligrams of protein ingredients were dissolved in 15 mL of Milli-Q water and homogenized at 12,000 rpm for 5 min (S Homogeniser, VWR). The foam volumes were recorded immediately (V_0_) and after 30 min (V_30_), respectively. FC and FS were determined using the following equation:

FC (%) = $\frac{V_{0}}{15}$ **×** 100 %

FS (%) = $\frac{V_{30}}{V_{0}}$ **×** 100 %

Where V__0__ is volume of foam just after homogenization (at 0 min) and V__30__ is volume of foam after 30 min.

#### 2.12.3 Emulsifying capacity and stability

Emulsifying capacity (EC) and stability (ES) of protein ingredients were evaluated using the method described in Boye et al. (2010a), with some modifications. Five millilitres of soybean oil were added to 15 mL of 0.5 % (w/v) protein ingredient solution prepared in Milli-Q water, with the pH adjusted to 7. The mixture was homogenized at 12,000 rpm for 2 min (S homogeniser, VWR). Fifty millilitres of emulsions were collected from the bottom of centrifuge tube immediately and after 10 min, respectively. The collected emulsion was diluted with 2 mL of 0.1 % (w/v) SDS. The absorbance of diluted emulsion solution was measured at 500 nm. EC and ESI were calculated according to the following equations:

EC (m^2^/g) = $\frac{2\times 2.303\times {Abs}_{0\;\min}\times dilution\;factor}{\left(1-\Phi \right)\times protein\;concentration\times 100}$

ESI (min) = $\frac{{Abs}_{0\;\min}\times 10}{\left({Abs}_{0\;\min-}{Abs}_{10\;\min}\right)}$

Where Abs_0min_ is the absorbance of the diluted emulsion collected immediately after homogenization, Abs_10 min_ is the absorbance of the diluted emulsion collected after 10 min, and Φ is oil volume fraction.

#### 2.12.4 Gelling properties

The least gelling concentration (LGC) of protein ingredients were determined using the method mentioned in de Paiva Gouvêa et al. (2023). Different amounts of protein ingredients (0.1 – 1g) were dissolved in 5 mL of Milli-Q water to make suspensions with concentrations ranging from 2 to 20 % (w/v). After vortexing for 30 s, the tubes (borosilicate glass tube, 25 mm in diameter and 150 mm in length, Z740968, Sigma) were heated in a boiling water bath for 1 h. The tubes were then rapidly cooled using running tap water. Gel formation in the suspensions was determined after the tube were placed at 4 °C overnight.

#### 2.12.5 Protein solubility

Protein solubility of protein ingredients was determined at different pH levels, ranging from 2 to 9, according to Boye et al. (2010a), with some modifications. Briefly, protein ingredients containing 100 mg of protein was dissolved in 20 mL of Milli-Q water, and the pH of the solution was adjusted to the desired value using 1M NaOH or 1M HCl. After shaking for 30 min at 200 rpm (Orbital Shaker 36508, Heidolph), the supernatant was collected following centrifugation at 3,500 rpm (ROTINA 380, Hettich) for 20 min. Soluble protein content in the supernatant was determined using Pierce™ BCA protein assay kit. Protein solubility at each pH level was calculated as the ratio (%) of protein in the supernatant to the total protein (100 mg) in the protein ingredients. Bovine serum albumin was used as standard, with concentrations ranging from 25 to 2,000 μg/mL.

#### 2.12.6 Particle size and zeta potential

Nanoparticle size of protein ingredients was measured using Mastersizer 3000 with the Aero S Dry powder dispersion unit. Zeta potential of protein ingredients was analysed using Zeta-sizer 3000. For zeta-potential measurement, 3 mg of protein ingredients were dissolved in 35 mL of Milli-Q water, and the pH of solution was adjusted to a range of 2–9.

### 2.13 Statistical analysis

All measurements were carried out in triplicate. Results were presented as the mean ± standard deviation, with all analyses conducted in triplicates. Statistical analysis was performed using GraphPad Prism 10 (GraphPad Software, Boston, MA, USA). Significantly differences among protein ingredients were analysed using Tukey's HSD and multiple *t*-test at a significance level of P-value < 0.05. Pearson correlation coefficient analysis and principal component analysis were performed using OrginPro 2021 software (OriginLab Crop., MA, USA)

## 3 Results and discussion

### 3.1 Protein content, total starch, available carbohydrate, total dietary fibre, and moisture content

As shown in Table 1, the crude protein content of fava bean, chickpea and red lentil are 19.17 g/100 g, 16.54 g/100 g, and 17.80 g/100 g, respectively. Ruckmangathan et al. (2022) reported a similar crude protein content (17.10 %) in chickpea, but a much higher crude protein content was found in fava bean (25.80 %) and red lentil (25.10 %). Other studies reported much higher crude content in legumes, for instance, the crude protein content of 15 fava bean cultivars ranged from 22.7 to 28.3 % (Labba et al., 2021). Qayyum et al. (2012) reported crude protein contents in chickpea and lentil were 22.83 % and 31.12 %, respectively. Moreover, Sánchez-Vioque et al. (1999) reported a crude protein content of 24.7 % for chickpea, while the ones in four varieties of red lentils ranged from 22.57 to 31.17 % (Wang, 2008). These findings highlighted that the crude protein content of legumes varied by location, cultivar, and plant growth stage (Wang and Daun, 2004). As expected, dry fractionation led to a significant increase in protein content across all three analysed legumes, reaching 58.13 g/100 g in fava bean, 41.41 g/100 g in chickpea, and 59.04 g/100 g in red lentil. Wet fractionation further enhanced the protein concentration, reaching 58.26 g/100 g in chickpea and up to 83.79 g/100 g in fava bean.

**Table 1:** Protein content (g/100 g dw), total starch content (g/100 g dw), available carbohydrate (g/100 g dw), total dietary fibre (g/100 g dw) and moisture (%) of fava bean, chickpea, and red lentil in three forms: raw flour, dry-fractionated protein-enriched fractions, and wet-fractionated protein-enriched fractions.

Sample	Processing	Protein content (g/100 g dw)	Total starch (g/100 g dw)^a^	Available carbohydrate (g/100 g dw)	Total dietary fibre (g/100 g dw)	Moisture (%)
Fava bean	**Raw**	19.17 ± 0.11^f^	60.75 ± 0.77^a^	45.22 ± 0.58^a^	10.20 ± 0.14^b^	9.53 ± 0.13^a^
	**Dry fractionation**	58.13 ± 0.10^d^	15.32 ± 0.41^e^	15.47 ± 0.30^d^	13.60 ± 0.38^a^	7.40 ± 0.23^c^
	**Wet fractionation**	83.79 ± 0.45^a^	0.55 ± 0.02^h^	3.91 ± 0.09^f^	4.48 ± 0.12^e^	6.09 ± 0.15^d^
						
**Chickpea**	**Raw**	16.54 ± 0.04^h^	50.26 ± 0.84^c^	22.33 ± 1.10^c^	10.12 ± 0.14^b^	7.33 ± 0.14^b^
	**Dry fractionation**	41.42 ± 0.38^e^	20.57 ± 0.92^d^	6.81 ± 0.41^e^	14.64 ± 1.86^a^	5.75 ± 0.05^d^
	**Wet fractionation**	58.26 ± 0.03^d^	0.57 ± 0.02^h^	1.01 ± 0.34^h^	3.04 ± 0.40^f^	5.12 ± 0.18^e^
						
**Red lentil**	**Raw**	17.80 ± 0.26^g^	57.38 ± 1.07^b^	30.14 ± 0.12^b^	6.74 ± 0.06^d^	7.82 ± 0.19^b^
	**Dry fractionation**	59.04 ± 0.19^c^	10.20 ± 0.21^f^	3.29 ± 0.12^g^	7.62 ± 0.06^c^	4.96 ± 0.16^e^
	**Wet fractionation**	80.55 ± 0.30^b^	1.38 ± 0.04^g^	0.00^i^	1.62 ± 0.08^g^	5.29 ± 0.21^e^

Data expressed as mean ± SD, n = 3. Different lowercase letters within each column indicate significant differences (p-value < 0.05).

a Total starch content in wheat starch control was 83.64 ± 0.75 g/100 g dw.

The starch content in these legumes decreased significantly during processing: initially ranging from 50.26 g/100 g in chickpea, 57.38 g/100 g in red lentil and 60.75 g/100 g in fava bean, it decreased to 20.57 g/100 g (chickpea), 15.32 g/100 g (fava bean) and 10.20 g/100 g (red lentil) after dry fractionation, and was further reduced to just 1.38 g/100 g (red lentil), 0.57 g/100 g (chickpea) and 0.55 g/100 g (fava bean) following wet fractionation. Schlangen et al. (2022) reported a similar trend in protein content of fine fractions obtained by dry fractionation. The protein content in mung bean, yellow pea and cowpea flours was around ∼23 %, which increased to ∼42 % – ∼58 % after dry fractionation. In addition, the protein content in the fine fraction of pea ranged from ∼50 % to ∼55 % after dry fractionation at different classifier speeds (5,000 rpm and 12,000 rpm), increasing from 23 % in unprocessed flours (Pelgrom et al., 2013). Dumoulin et al. (2021) applied dry fractionation to fava bean flour, enriching the protein content from 27.7 to 53.6 %. In their study, wet fractionation was applied to a coarse starch-rich fraction, resulting in a significant increase in protein content, which rose from 22.2 % to 60.6 %. For studies that applied wet fractionation directly to raw flour, Ruiz-Ruiz et al. (2012) reported a protein level of 73.03 % in hard-to-cook black bean and 68.83 % in freshly harvested bean after wet fractionation. Higher protein contents were found in commercial wet-fractioned fava bean and chickpea, which were 86.9 % and 82.9 %, respectively (Li et al., 2024). These findings support the conclusion that wet fractionation is more effective in enriching protein content. In dry fractionation, water-soluble protein fragments were not fully disentangled from starch granules and were ultimately transferred to the coarse fraction rather than the fine fraction (Möller et al., 2021). Additionally, it is important to highlight that, following both dry and wet fractionation, the protein content in chickpea remained significantly lower than that in fava bean and red lentil. This discrepancy may be attributed to the stronger attachment of protein particles to starch granules in chickpea, which likely reduced separation efficiency during air classification and protein isoelectric point precipitation (Schlangen et al., 2022). Consequently, the final protein content in chickpea-enriched fractions remained lower than that of the other legumes after processing.

Available carbohydrate contents of fava bean, chickpea, and red lentil were 45.22, 22.33 and 30.14 g/100 g, respectively. The difference between total starch and available carbohydrate was largely attributed to the presence of resistant starch. As reported by García-Alonso et al. (1998), resistant starch is abundant in legumes, ranging from 16.1 % to 21.3 %. Similarly, Bozkır et al. (2023) claimed that the average resistant starch content of 62 common bean varieties was 16.41 ± 12.77 %. After dry fractionation, available carbohydrate content was largely reduced (ranging from -15.52 g/100 g to -29.75 g/100 g), due to the efficient removal of starch. After wet fractionation, only minimal amounts of available carbohydrates remained present, ranging from 0 to 3.91 g/100 g.

In terms of total dietary fibre (TDF) content, no significant difference was observed between fava bean (10.20 g/100g) and chickpea (10.12 g/100 g). However, red lentil demonstrated a significantly lower fibre content (6.74 g/100 g). Millar et al. (2019) reported a slightly higher TDF content in fava bean at 13.80 g/100g, while Costantini et al. (2021) reported a lower value of 8.40 g/100 g. The TDF content in chickpea observed in the present study was lower than those reported by Sreerama et al. (2012), who found a TDF content of 14.8 g/100 g. Furthermore, Ajay et al. (2024) also reported a higher TDF range for chickpea, ranging from 18.74 g/100 g – 21.86 g/100 g. For red lentil, the TDF value in this study aligns with the range reported by Wang (2008), who observed TDF contents of four red lentil varieties ranging from 3.5 g/100 g to 7.4 g/100 g. Dry fractionation significantly increased TDF content in all legume flours. This effect is likely because protein bodies are surrounded with fibre-rich cell walls (Wockenfuss et al., 2023). Consequently, flours tend to exhibit higher TDF because dry fractionation concentrate proteins that are associated with fibre. De Angelis et al. (2022) also reported an increase of TDF in legume-based pasta formulated with yellow lentils and whole rice (90:10 w/w), where the fibre content rose from 6.17 g/100 g – 6.63 g/100 g after dry fractionation. Similarly, Pelgrom et al. (2015a) observed a noticeable increase in fibre content of yellow pea after dry fractionation, from 26.1 g/100 g – 42.0 g/100 g. Furthermore, Li et al. (2024) reported that TDF in fava bean and yellow pea increased from 7.3 g/100 g and 9.8 g/100 g – 16.1 g/100 g and 20.1 g/100 g, respectively, after dry fractionation. However, they also highlighted a huge reduction in TDF after wet fractionation, with values decreasing to 3.1 g/100 g for fava bean, and 3.4 g/100 g for yellow pea. These findings were consistent with the results of this study, where significantly reductions in TDF were observed in fava bean (-5.72 g/100 g), chickpea (-7.08 g/100 g), and red lentil (-5.12 g/100 g) after wet fractionation. This suggests that a large proportion of dietary fibre was removed during the protein extraction step.

The moisture content of fava bean, chickpea, and red lentil flours were 9.53 %, 7.33 %, and 7.83 %, respectively. These values were slightly lower than those reported by Skylas et al. (2023), who observed moisture content of 10.9 % – 11.7 % for fava bean flour and 9.8 % – 10.0 % for red lentil flour. Similarly, Jagannadham et al. (2014) reported a higher moisture content of 9.35 % for chickpea flour. Ozolina et al. (2024) presented comparable values, a moisture content range of 7.47 % – 7.79 % for red lentil flour and 8.53 % – 8.75 % for fava bean flour. The slight difference observed in moisture content could be attributed to variations in legume growth conditions and differences in milling technology, such as milling speed (Pelgrom et al., 2013; Skylas et al., 2023). It was evident that dry fractionation significantly reduced the moisture content, with reductions ranging from -1.58 % to -2.86 %. Dry fractionation did not directly remove water from protein-enriched flours but instead relied on the separation of drier protein-enriched fractions from relatively heavier and higher moisture starch-rich fractions (Xing, 2021). The wet fractionation process was also found to reduce the moisture content, due to the spray drying step that is performed to obtain the dry ingredients. This process typically resulted in a moisture level below 5 % in the final product (Tontul and Topuz, 2017), which was only slightly different from the moisture levels measured in this study, ranging from 5.12 % to 6.09 %.

### 3.2 Antinutritional factors

Antinutritional factors are widely present in plants and comprise phytochemicals or secondary metabolites that protect plants from damage caused by insects, herbivores, and inherent pathogens (Prajapati et al., 2021). During dry and wet fractionation, the protein concentration in processed flour increases, leading to changes in the content of antinutritional factors (Amin et al., 2022). These compounds negatively impact the bioaccessibility and bioavailability of essential nutrients, such as protein, minerals, and vitamins (Soni et al., 2022). However, it is important to mention that antinutritional factors may also confer significant health benefits, such as antioxidant activity, prevention of type 2 diabetes, anti-inflammatory effects, and anticancer properties (Manzanilla-Valdez et al., 2024c). In this study, several antinutritional factors, including polyphenols, phytic acid, condensed tannins, saponins, and trypsin inhibitors, were quantified in legume flours before and after dry and wet fractionation. The results are presented in Table 2.

**Table 2:** Total polyphenols (mg GAE/per 100g dw), phytic acid (g/100g dw), condensed tannins (mg/100g dw), saponins (mg/100g dw) and trypsin inhibitors (TUI/mg dw) of fava bean, chickpea, and red lentil in three forms: raw flour, dry-fractionated protein-enriched fractions, and wet-fractionated protein-enriched fractions.

Sample	Processing	Total polyphenols (mg GAE/per 100 g dw)	Phytic acid (g/100g dw)	Condensed tannins (mg/100 g dw)	Saponins (mg/100 g dw)	Trypsin inhibitors (TUI/mg dw)
**Fava bean**	**Raw**	263.8 ± 13.0^c^	1.224 ± 0.057^c^	10.94 ± 0.88^f^	584.5 ± 34.6^g^	0.629 ± 0.059^d^
	**Dry fractionation**	568.9 ± 28.1^a^	0.854 ± 0.055^e^	55.18 ± 2.59^c^	946.5 ± 37.7^d^	1.451 ± 0.044^b^
	**Wet fractionation**	425.7 ± 22.5^b^	0.797 ± 0.050^e^	38.76 ± 1.60^d^	1237.9 ± 59.7^c^	1.395 ± 0.102^b^
						
**Chickpea**	**Raw**	110.3 ± 8.0^e^	1.114 ± 0.058^cd^	21.47 ± 0.9^e^	807.4 ± 48.0^e^	0.287 ± 0.008^e^
	**Dry fractionation**	259.8 ± 15.6^c^	2.279 ± 0.131^a^	62.18 ± 2.12^b^	1616.2 ± 69.3^a^	0.968 ± 0.038^c^
	**Wet fractionation**	252.7 ± 9.1^c^	1.914 ± 0.069^b^	62.50 ± 3.06^b^	1661.6 ± 59.4^a^	1.312 ± 0.090^b^
						
**Red lentil**	**Raw**	91.1 ± 7.4^f^	1.075 ± 0.043^d^	19.85 ± 0.76^e^	714.0 ± 30.5^f^	0.602 ± 0.013^d^
	**Dry fractionation**	171.9 ± 10.4^d^	1.952 ± 0.068^b^	81.44 ± 3.47^a^	1312.6 ± 71.9^b^	2.287 ± 0.128^a^
	**Wet fractionation**	175.6 ± 7.5^d^	1.884 ± 0.097^b^	55.12 ± 2.64^c^	1395.7 ± 35.3^b^	1.009 ± 0.100^c^

Data expressed as mean ± SD, n = 3. Different lowercase letters within each column indicate significant differences (p-value < 0.05).

**Polyphenols** are well-documented bioactive compounds. However, they are considered as antinutritional factors. This is because polyphenols exhibited a strong affinity for proteins, which interacts with sulfhydryl groups and free amino acids, and consequently decreases protein digestibility and bioavailability of amino acids (Sęczyk et al., 2019). The highest total phenolic content (TPC) was found in fava bean flour (263.8 mg GAE/per 100g), followed by chickpea (110.3 mg GAE/per 100g), and red lentil (91.1 mg GAE/per 100g) flour. Labba et al. (2021) measured TPC in 15 fava bean varieties, values ranging from 140 mg GAE/per 100g to 500 mg GAE/per 100g, with the cultivar *Fernando* showing a similar TPC value of 230 mg GAE/per 100g. Meanwhile, Bubelova et al. (2018) reported comparable TPC values for dehulled red lentils (84.69 mg GAE/per 100g). The range of TPC values in different chickpea genotypes was 72 mg GAE/per 100g to 191 mg GAE/per 100g (Yadav et al., 2024), which is in agreement with the value reported in this study. However, several studies have reported significantly higher TPC values. Millar et al. (2019) reported a TPC value of 387.5 mg GAE/per 100g in fava bean. Saleh et al. (2019) observed much higher TPC values in chickpea (568 mg GAE/per 100g) and lentil (521 mg GAE/per 100g) flours. Skylas et al. (2023) found that TPC in faba bean and red lentil were 276 GAE/per 100g and 368 mg GAE/per 100g, respectively.

Fractionation methods significantly increased the TPC among all three legume flours, ranging from +61.37 % to +135.54 %. Wet-fractionated fava bean PF (425.7 mg GAE/per 100g) showed a lower TPC compared to that produced by dry fractionation (568.9 mg GAE/per 100g), while no significant difference was observed for the other two legumes. A significant increase in TPC (∼55 %) in fava bean after dry fractionation was also reported by Dumoulin et al. (2021). Similarly, after dry fractionation, TPC in fava bean, red lentil and yellow pea, increased by 145.94 %, 92.53 %, and 139.71 %, respectively (Skylas et al., 2023). Regarding wet fractionation, Shi et al. (2022) applied wet extraction and observed increases in TPC of Fabelle fava bean (+72.3 %), Malik fava bean (+72.4 %), Snowbird fava bean (+17.8 %), pea (+184.4 %), and soy (+23.7 %) respectively. However, no significant difference was found between defatted peanut flour (6 mg GAE/per 100g) and wet-extracted peanut protein concentrate (6 mg GAE/per 100g) (Asen et al., 2021).

**Phytic acid**, also known as myo-inositol hexakisphosphate, is responsible for reducing the absorption rate and bioavailability of metal ions, including zinc, iron, magnesium, and calcium, which can lead to mineral deficiencies (Samtiya et al., 2020). Phytic acid has been found in a wide range of legumes. Shi et al. (2018) reported different phytic acid levels among legumes, including fava beans (1.965 – 2.285 g/100g), common beans (1.564 – 1.882 g/100g), lentils (0.856 – 1.556 g/100g), chickpea (1.133 – 1.400 g/100g), and peas (0.855 – 1.240 g/100g). In this study, phytic acid values in chickpea (1.114 g/100g) and red lentil (1.075 g/100g) were similar, while a higher phytic acid level was detected in fava bean (1.224 g/100g). Similar phytic acid levels in fava beans have been reported by (Zehring et al., 2022), with values ranging from 0.80 to 1.37 g/100g. Meanwhile, Lazarte et al. (2015) also reported similar values of phytic acid in fava beans (1.170g/100g) and lentils (0.846 g/100g).

The impact of fractionation largely depended on the type of legumes. Phytic acid decreased in fava bean after dry (-30.23 %) and wet (-34.89 %) fractionation. In contrast, Dumoulin et al. (2021) reported that phytic acid in fava bean increased by ∼145.5 % after dry fractionation. In this study, the decrease in phytic acid after dry or wet fractionation might be attributed to processes such as milling and aqueous extraction. Milling removes the seed coat, where phytic acid is mainly concentrated (Feizollahi et al., 2021). In addition, aqueous extraction led to the dissolution and removal of phytic acid at acidic pH during wet fractionation. However, this phenomenon was observed only in fava beans. Phytic acid levels in chickpea increase by +104.58 % and +71.81 % following dry and wet fractionation processes, respectively. Similarly, in red lentil, the increases were +81.58 % after dry fractionation and +75.26 % after wet fractionation (Bloot et al., 2023). Similarly, De Angelis et al. (2021) observed an increase in total phytates in red lentils (+35.61 %), yellow lentils (+90.26 %), green peas (+60.34 %), and kabuli chickpeas (+78.61 %) after dry fractionation. Among five commercial hemp protein concentrates, those subjected to both dry and wet fractionation showed lower phytic acid levels (1.9 g/100 g, 1.6 g/100 g, and 1.3 g/100 g) compared to those that underwent dry fractionation only (3.4 g/100 g and 3.6 g/100 g) (Nasrollahzadeh et al., 2022). This tendency was also observed in fractionated chickpea PF, while fava bean and red lentil showed no significant difference in phytic acid levels between dry and wet fractionation.

**Condensed tannins (CT)** are astringent and polyphenolic compounds with a bitter taste. They form insoluble complexes with proteins and other organic compounds, including amino acids and alkaloids, which reduces their digestibility and absorption (Manzanilla-Valdez et al., 2024c). CT inhibit the activities of trypsin, chymotrypsin, amylase, and lipase, and interferes with dietary iron absorption. In animal studies, CT showed a negative impact on feed intake and growth rates (Gemede and Ratta, 2018). CT levels in legumes have been extensively quantified, for example, Baginsky et al. (2013) measured the CT content in 10 varieties of fava beans and found that the CT levels ranged from 30.9 mg/100 g to 95.88 mg/100 g. CT content in chickpea was reported as 175.23 mg/100 g by Dida Bulbula and Urga (2018). Similarly, Adamidou et al. (2011) also reported CT levels of 1.28g/100g in fava bean and 0.49 g/100 g in chickpea. Moreover, Zhang et al. (2015) observed that the TC content in red lentils across 10 cultivars, ranged from 300 mg/100 g to 582 mg/100 g. In this present study, CT levels in fava bean, chickpea and red lentil were found to be 10.94 mg/100 g, 21.47 mg/100 g, and 19.85 mg/100 g, respectively. These notably low CT values may be attributed to dehulling. Alonso et al. (2000) reported that CT content in dehulled fava bean was 15 mg/100 g, compared to 195 mg/100g in raw seeds. Similarly, Bautista-Expósito et al. (2022) observed a CT level of 1,583 mg/100 g in red lentil hulls, indicating that dehulling significantly reduces tannin levels in red lentils.

This study is the first to demonstrate that fractionation increased CT levels in three legumes. The increasing trend of CT in fava bean and chickpea was closely associated with TPC. However, in red lentils, the increase in CT content increased by dry fractionation (+310.28 %) was higher than that produced through wet fractionation (+177.68 %).

**Saponins** are steroid or triterpenoid glycosides. Due to bitter taste and throat-irritating activity, saponins led to decreased food intake and impaired growth. They also decrease the activity of digestive enzymes, destroy red blood cells, and negatively affect nutrient absorption (Gemede and Ratta, 2018). Legumes have been reported to be rich in saponins. Fenwick and Oakenfull (1983) reported that the saponin content in chickpea, fava bean, red lentil (small) and red lentil (large) were 5.6 g/100 g, 30 mg/100 g, 460 mg/100 g and 370 mg/100 g, respectively. Srivastava and Vasishtha (2013) observed relatively lower saponin contents in ten chickpea cultivars, ranging from 654.5 mg/100 g to 843.0 mg/100 g. In addition, Sharma and Sehgal (1992) reported a higher saponin content in two varieties of fava bean, with values of 1,370 mg/100 g and 1,331 mg/100 g. Moreover, Sharma et al. (2023) reviewed the saponin content in legumes reported in the literature and found that the total saponins (%) in lentils varied from 11 mg/100 g to 51 mg/100 g, while in chickpeas, it ranged from 150 mg/100 g to 600 mg/100 g. In this study, red lentil showed a relatively high saponin content (714.0 mg/100 g). While saponin content in chickpea (807.4 mg/100 g) and fava bean (584.5 mg/100 g) falls within the range reported above.

Despite extensive research on saponins in plant-based foods, there remains a limited systematic comparison of the impact of dry and wet fractionation methods on saponin levels in legumes. Similar to other antinutritional factors, fractionation processes markedly increased the saponin concentrations. Between the two fractionation methods, a significant difference was observed only in fava beans, where wet-fractionated fava bean PF exhibited higher saponin content (1,237.9 mg/100 g) compared to dry-fractionated PF (946.5 mg/100 g). Similar, saponin content in red quinoa flour were 9,680 mg/100g, and increased to 16,220 mg/100 g after protein enrichment by wet fractionation (Manzanilla-Valdez et al., 2024a, 2024b). Fenwick and Oakenfull (1983) reported saponin contents of 430 mg/100g in raw fava bean and 820 mg/100g in protein isolate, supporting the notion that protein-enrichment processes lead to a significant increase in saponin concentration.

**Trypsin inhibitors (TI)** are considered as antinutritional factors because they directly inhibit the key digestive proteases, including trypsin and chymotrypsin, thereby reducing protein digestion and absorption (Manzanilla-Valdez et al., 2024c). In this study, no significant difference was found in TI activity between fava bean (0.629 TIU/mg) and red lentil (0.602 TIU/mg), while chickpea showed a significantly lower value at 0.287 TIU/mg. Much higher TI activity for these legumes has been previously reported. For instance, Vidal-Valverde et al. (1997) reported that the TI activity of fava bean was 2.62 TIU/mg. Labba et al. (2021) provided a range of TIU values for 15 different cultivars of fava bean, which ranged from 1.2 TIU/mg to 23.1 TIU/mg. In addition, the TI activity for Desi (16 cultivars) and Kabuli (21 cultivars) chickpeas ranged from 3.14 TIU/mg to 15.06 TIU/mg and from 3.48 TIU/mg to 18.31 TIU/mg, respectively (Rincón et al., 1998). Regarding red lentils, Barbana and Boye (2013) observed that TI activity was 0.94 TIU/mg. Ruckmangathan et al. (2022) reported a similar TI value for red lentils (0.96 TIU/mg). They also reported a TI value of 0.68 TIU/mg for fava bean, which was found to be similar to the value reported in this study.

Dry fractionation increased the TI activity in fava bean (+130.68 %), chickpea (+237.28 %) and red lentil (+279.90 %). Dumoulin et al. (2021) also found that the TI activity of fava bean increased after dry fractionation, but only slightly raised from ∼10.4 TIU/mg to ∼15 TIU/mg. Similarly, Vogelsang-O’Dwyer et al. (2020) reported a 64.8 % increase of TI activity in fava bean after dry fractionation. However, they observed that the TI activity decreased significantly after isoelectric precipitation (wet fractionation), from 1.42 TIU/mg (raw fava bean flour) to 0.29 TIU/mg, which differed with the findings in the current study. In this study, wet-fractionated legume PF exhibited higher TI activity compared to unprocessed flours, with increases ranging from +67.61 % to +357.14 %. When compared to those produced by dry fractionation, the changes were sample-dependent. Specifically, TI activity increased in chickpea (+36.5 %), decreased in red lentil (−56.0 %), and showed no significant change in fava bean. Mondor et al. (2009) found a slight increase in TIU of kabuli chickpea (from 20.60 to 21.00 TIU/mg), but no change in Desi chickpea after isoelectric precipitation. de Paiva Gouvêa et al. (2024) also reported no significant differences were found in TI activity between fava bean flour and fava bean protein concentrate (obtained by isoelectric precipitation). The contrasting trend in TI activity observed in this study may be attributed to the fact that only trypsin inhibitors interacting with protein bodies were retained, while those bound to other areas in the seeds, such as seed coat and embryonic axis, were removed (Wang et al., 2023b). This explained the lower TIU values found in these unprocessed legumes studied and the increase in TI activity after protein enrichment processes.

### 3.3 Nutritional properties

**Fig. 1:**
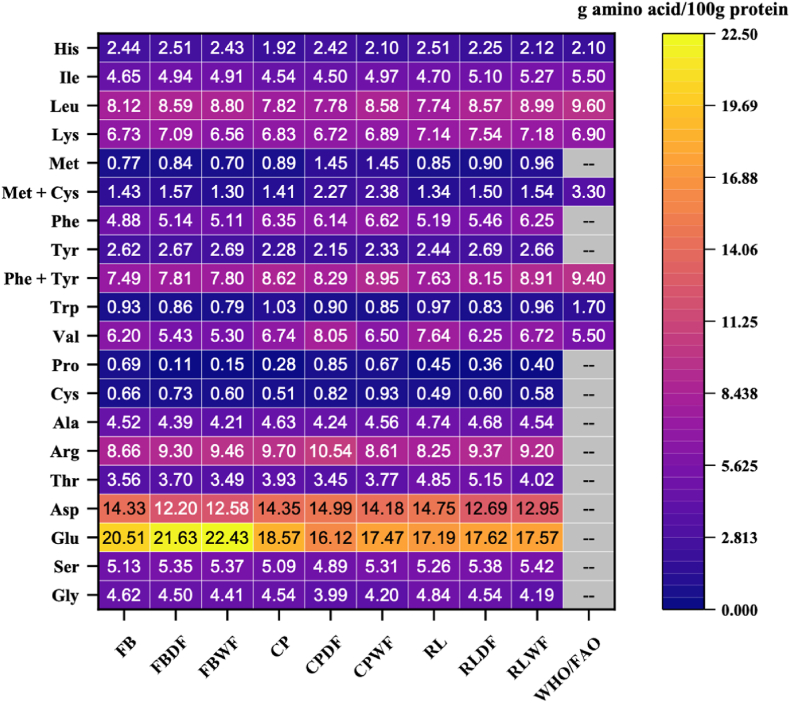
Amino acid profiles (g amino acid/100g protein) of fava bean, chickpea, and red lentil in three forms: raw flour, dry-fractionated protein-enriched fractions, and wet-fractionated protein-enriched fractions. The FAO/WHO infant pattern (2013) was used as a reference. The change in colour of the scale from blue to red indicate the amino acid content from low to high. Gly, glycine; Lys, lysine; Glu, glutamine; Ser, serine; Ala, alanine; Leu, leucine; Met, methionine; Phe, phenylalanine; Trp, tryptophan; Pro, proline; Val, valine; Ile, isoleucine; Cys, cysteine; Tyr, tyrosine; His, histidine; Arg, arginine; Asn, asparagine; Asp, aspartic acid; Thr, threonine. Data presented as mean, n = 3, (p < 0.05). FB, raw fava bean flour; FBDF, fava bean protein-enriched ingredient after dry fractionation; FBWF, fava bean protein-enriched ingredient after wet fractionation; CP, raw chickpea flour; CPDF, chickpea protein-enriched ingredient after dry fractionation; CPWF, chickpea protein-enriched ingredient after wet fractionation; RL, raw red lentil flour; RLDF, red lentil protein-enriched ingredient after dry fractionation, and RLWF, red lentil protein-enriched ingredient after wet fractionation.

The amino acid (AA) composition of fava bean, chickpea, and red lentil is presented in Fig. 1. Significant statistic differences were observed in the AA profiles of these unprocessed flours. However, considerable similarities were also evident, reflecting that they are all from the Fabaceae family. Glu was the most abundant amino acid in all three flours, ranging from 17.19 g/100 g to 20.51 g/100 g. Asp was the second most prevalent amino acid (14.33 g/100 g–14.75 g/100 g), followed by Arg (8.25 g/100 g – 9.70 g/100 g). In contrast, Pro was the least abundant amino acid, the highest level of this amino acid was found in fava bean, at 0.69 g/100 g. Furthermore, Cys (0.49 g/100 g – 0.66 g/100 g), Met (0.77 g/100 g – 0.85 g/100 g), and Trp (0.93 g/100 g – 1.03 g/100 g) were identified as limiting AAs. The AA composition reported in the present study largely aligned with previous literature (Labba et al., 2021; Lee et al., 2021; Sánchez-Velázquez et al., 2021). The minor variations detected among studies can be attributed to differences in legume variety and growth conditions (Boye et al., 2010b).

Fractionation methods were found to significantly but minimally affect the AA composition. Dry fractionation led to a slight increase in essential AAs, ranging from +0.42 % to +1.71 %. Wet fractionation had no significant effect on the essential AA profiles of fava bean flour but resulted in an increase of +1.89 % and +1.93 % in essential AA content for chickpea and red lentil, respectively. Li et al. (2024) compared fava bean and yellow pea flours obtained through dry and wet fractionation and found that the proportions of essential AAs were similar, 37.6 % vs. 38.0 % for fava bean and 38.9 % vs. 38.7 % for yellow pea. Lys was abundant in all legume flours, ranging from 6.73 g/100 g – 7.14 g/100 g. Despite Lys has been well-documented as heat sensitive (Hendriks, 2018), both dry and wet fraction had minimal impact on Lys content. The high Lys content in legumes made them an excellent candidate for blending with cereals to provide a balanced amino acid composition (Herreman et al., 2020).

It can be concluded that fractionation methods had a limited impact on AA composition of fava bean, chickpea, and red lentil. However, further investigation is required to evaluate the effects of both protein-enrich techniques on nutritional quality of legumes. This is because nutritional quality is not only determined by AA composition, but also by digestion and absorption, which are essential to meet the dietary AA requirements (Boye et al., 2010b). Therefore, additional parameters, including AAS (%), EAAI, BV, PER_1-5_, and IVPD (%) and IVPDCAAS have been calculated and are presented in the following section.

Amino acid score (AAS) is an essential parameter used to evaluate the adequacy of protein sources to supply essential AAs relative to the reference pattern values (FAO/WHO, 1991). The AAS values of most limiting essential AAs are summarised in Table 3. Among the raw legume flours, the AAS of Met + Cys was the lowest, ranging from 54.56 % to 57.26 %, indicating that Met + Cys were the most limiting amino acids. This observation aligned with Swanson (1990), who reported that both sulphur-containing AAs, together with Trp, were generally low in pulses. After fractionation processing, Met **+** Cys remained the most limiting amino acids in fava bean and red lentil. However, in chickpea, the most limiting AA shifted to Trp, with AAS of 82.10 % and 77.34 % after dry and wet fractionation, respectively. Simultaneously, AAS for Met **+** Cys in chickpea significantly increased to 90.74 % and 95.37 % under the same conditions. This indicates a notable improvement in nutritional quality of chickpea protein. In contrast, a reduction in AAS was observed in fava bean after wet fractionation, where AAS for Met **+** Cys decreased to 52.04 %. This was the only case in this study where protein enrichment had a negative impact on AAS of the most limiting essential AAs.

**Table 3:** Protein quality parameters of fava bean, chickpea, and red lentil in three forms: raw flour, dry-fractionated protein-enriched fractions, and wet-fractionated protein-enriched fractions.

Sample	Processing	AAS^a^ (%)	EAAI^b^ (%)	BV^c^	PER_1_^d^	PER_2_^d^	PER_3_^d^	PER_4_^d^	PER_5_^d^	IVPD^e^ (%)	IVPDCAAS^f^ (%)
**Fava bean**	**Raw**	57.26 (Met + Cys)	70.92	65.61	2.99	2.95	3.01	2.71	2.92	79.84 ± 1.74^bc^	45.72
	**Dry fractionation**	62.93 (Met + Cys)	72.22	67.02	3.23	3.15	3.36	2.78	3.02	82.01 ± 0.93^ab^	51.61
	**Wet fractionation**	52.04 (Met + Cys)	68.70	63.18	3.32	3.24	3.43	2.71	2.97	84.12 ± 0.36^a^	43.78
											
**Chickpea**	**Raw**	56.22 (Met + Cys)	71.91	66.69	2.87	2.84	3.15	2.89	3.07	78.87 ± 2.04^bc^	44.34
	**Dry fractionation**	82.10 (Trp)	76.49	71.57	2.82	2.84	3.47	2.97	3.21	76.04 ± 1.99^c^	62.43
	**Wet fractionation**	77.34 (Trp)	76.67	71.87	3.20	3.18	3.93	3.03	3.12	76.64 ± 0.46^c^	59.38
											
**Red lentil**	**Raw**	53.56 (Met + Cys)	75.50	70.59	2.82	2.79	2.91	2.97	3.09	80.08 ± 0.91^b^	42.89
	**Dry fractionation**	60.00 (Met + Cys)	75.65	70.75	3.21	3.14	3.35	3.04	3.21	78.75 ± 1.81^bc^	47.25
	**Wet fractionation**	61.79 (Met + Cys)	76.15	71.31	3.39	3.33	3.72	3.07	3.22	84.06 ± 1.52^a^	51.94

Different lowercase letters within the IVPD (%) column indicate significant differences (p-value < 0.05), data expressed as mean ± SD, n = 3.

Note: EAAI (%), AAS, BV (%), PER_1–5_ and IVPDCAAS (%) are calculated values, no standard deviation is available.

a Amino acid score.

b Essential amino acid index (EAAI) on total amino acids (TAA).

c Biological value.

d Protein efficiency ratio.

e *In vitro* protein digestibility.

f *In vitro* protein-digestibility corrected amino acid score.

EAAI evaluates protein quality by assessing the profiles of all essential amino acids, rather than individually, using egg protein as the reference (Oser, 1959). A higher EAAI is directly associated with a more balanced amino acid composition, improved protein quality, and enhanced protein efficiency. The EAAI of the three legumes flours ranged from 70.92 % to 75.50 %, classifying them as useful protein sources (70 < EAAI <80) (Chang et al., 2023). In most cases, fractionation had a positive impact on EAAI, particularly in chickpea, where an increased Met + Cys content resulted in EAAI values of 76.49 % after dry fractionation and 76.67 % after wet fractionation. However, a negative impact was observed in fava bean where a reduction in Met + Cys content lead to a decrease in EAAI to 68.70 % following wet fractionation, a value considered inadequate in terms of nutritional quality (EAAI <70) (Chang et al., 2023). BV is another parameter associated with the proportion of absorbed protein that is utilized by the human body. Protein sources with both EAAI and BV greater than 70 are considered nutritionally valuable and are effectively absorbed and metabolized (Mir et al., 2019). In this study, red lentil flour was identified as a promising protein source, with an initial BV of 70.59. Fractionation slightly improved its nutritional quality, leading to increased BV values of 70.75 and 71.31 after dry and wet fractionation, respectively. However, BV for fava bean flour, particularly wet-fractionated fava bean PF remained low (63.18), thereby additional strategies are required to enhance its amino acid profile. One potential approach is blending fava bean flour with cereals to achieve a more balanced AA composition, thereby improving both EAAI and BV. PER is defined as the weight gain per unit of protein consumed in animal studies and is commonly used to assess the ability of a protein to support animal growth. In this study, the PER values for all unprocessed flours and fractionated PF exceeded 2.7, indicating their suitability as excellent protein sources for animal consumption (Hoffman and Falvo, 2004). A slight decrease in PER was observed in chickpea after dry fractionation, with values decreasing from 2.87 to 2.82. Aside from this, all other fractionation treatments positively influenced PER, with wet fractionation demonstrating the most significant improvements (27.8 %). These results suggest that all legume flours analysed effectively support growth in animal models. Nevertheless, further research is needed to determine human growth requirements, as the correlation between amino acid profiles that support growth in humans and animals remains weak (Adhikari et al., 2022; Deglaire and Moughan, 2012; Hoffman and Falvo, 2004).

IVPD is another critical protein quality parameter that provides insights into protein bioavailability. As shown in Table 3, the IVPD values for fava bean, chickpea, and red lentil were 79.84 %, 78.87 %, and 80.08 %, respectively. The similar digestibility values observed among these legumes may be attributed to their comparable amino acid profiles and storage proteins. Previous studies have reported slightly lower IVPD values, with Olakanmi et al. (2024) measuring IVPD at ∼75 % for fava bean flour, and Portari et al. (2005) reporting 72.36 % for chickpea flour. Similarly, Barbana and Boye (2013) documented an IVPD of 77.05 % for red lentil flour. The slightly higher IVPD values observed in this study may be attributed to low levels of trypsin inhibitors. Interestingly, dry fractionation did not improve IVPD, whereas a significant increase was observed in fava bean (from 79.84 % to 84.12 %) and red lentil (from 80.08 % to 84.06 %) following wet fractionation. Fractionation has been shown to enrich protein content and improve accessibility to larger particles, thereby contributing to an increase in protein digestibility (Pelgrom et al., 2014). However, the accumulation of anti-nutritional factors following both dry and wet fractionation negatively impact protein digestibility. Particularly, in wet fractionation, protein denaturation and subsequently aggregation further reduced protein digestibility (Zhang et al., 2023). Consequently, the overall improvement in IVPD was limited.

**Table 4:** Secondary structure and surface hydrophobicity of fava bean, chickpea, and red lentil in three forms: raw flour, dry-fractionated protein-enriched fractions, and wet-fractionated protein-enriched fractions.

Sample	Processing	β-sheet (%)	Random coil (%)	α-helix (%)	β-turn (%)	Surface hydrophobicity (H_0_)
**Fava bean**	**Raw**	27.68 ± 3.25^c^	34.80 ± 3.44^a^	29.05 ± 0.50^a^	8.47 ± 1.06^c^	44,243 ± 3,399^c^
	**Dry fractionation**	36.25 ± 2.37^ab^	33.49 ± 1.01^a^	22.45 ± 2.14^b^	7.82 ± 0.77^c^	29,058 ± 1,279^d^
	**Wet fractionation**	38.29 ± 0.43^ab^	29.57 ± 2.77^a^	22.80 ± 2.09^b^	9.33 ± 1.56^c^	62,739 ± 3,455^b^
						
**Chickpea**	**Raw**	40.33 ± 1.53^a^	30.02 ± 2.43^a^	18.63 ± 0.62^bc^	11.01 ± 3.97^c^	26,001 ± 2,348^e^
	**Dry fractionation**	34.18 ± 2.17^b^	22.91 ± 1.04^b^	24.45 ± 1.05^bc^	18.46 ± 2.07^b^	35,204 ± 1,104^cd^
	**Wet fractionation**	37.07 ± 1.49^ab^	34.37 ± 2.21^a^	21.46 ± 0.55^bcd^	7.10 ± 0.51^c^	92,915 ± 6,601^a^
						
**Red lentil**	**Raw**	27.97 ± 0.54^c^	20.57 ± 2.16^b^	22.60 ± 0.94^bcd^	28.85 ± 3.27^a^	32,492 ± 3,059^d^
	**Dry fractionation**	34.82 ± 0.30^b^	22.65 ± 0.50^b^	24.83 ± 0.57^bc^	17.71 ± 0.57^b^	36,307 ± 1,519^cd^
	**Wet fractionation**	40.48 ± 0.65^a^	30.56 ± 1.83^a^	19.07 ± 2.05^d^	9.88 ± 2.05^c^	99,381 ± 3,226^a^

Data expressed as mean ± SD, n = 3. Different lowercase letters within each column indicate significant differences (p-value < 0.05).

**Table 5:** Water holding capacity (g/g), oil holding capacity (g/g), foaming capacity (%), foaming stability (%), emulsifying activity index (m^2^/g), and emulsifying stability index (min) of fava bean, chickpea, and red lentil in three forms: raw flour, dry-fractionated protein-enriched fractions, and wet-fractionated protein-enriched fractions.

Ingredients	Processing	Water holding capacity (g/g)	Oil holding capacity (g/g)	Foaming capacity (%)	Foaming stability (%)	Emulsifying activity index (m^2^/g)	Emulsifying stability index (min)
**Fava bean**	**Raw**	1.08 ± 0.02^f^	3.45 ± 0.19^bc^	115.6 ± 3.8^c^	71.2 ± 0.6^d^	52.2 ± 1.9^f^	30.9 ± 1.7^e^
	**Dry fractionation**	1.37 ± 0.03^e^	3.30 ± 0.26^cd^	85.6 ± 3.7^e^	73.7 ± 1.7^cd^	63.6 ± 1.6^e^	31.9 ± 1.8^de^
	**Wet fractionation**	2.33 ± 0.02^b^	2.78 ± 0.12^e^	139.1 ± 1.0^a^	49.2 ± 1.0^e^	94.9 ± 4.0^c^	24.3 ± 2.0^f^
							
**Chickpea**	**Raw**	1.71 ± 0.09^d^	3.40 ± 0.07^c^	50.9 ± 1.7^g^	93.1 ± 1.8^a^	67.9 ± 1.4^de^	65.4 ± 5.7^b^
	**Dry fractionation**	1.76 ± 0.05^d^	3.98 ± 0.21^a^	76.4 ± 3.1^f^	83.4 ± 1.1^b^	99.8 ± 4.2^bc^	172.3 ± 21.0^a^
	**Wet fractionation**	2.21 ± 0.04^b^	3.16 ± 0.04^d^	0^h^	N.D.^a^	95.5 ± 5.1^c^	52.2 ± 4.0^c^
							
**Red lentil**	**Raw**	1.26 ± 0.01^e^	3.17 ± 0.16^cd^	113.3 ± 0.7^c^	71.0 ± 1.1^d^	75.2 ± 2.3^d^	33.6 ± 0.8^d^
	**Dry fractionation**	2.03 ± 0.05^c^	3.88 ± 0.17^ab^	92.7 ± 2.0^d^	75.3 ± 2.1^c^	109.3 ± 1.9^a^	57.6 ± 3.9^bc^
	**Wet fractionation**	2.83 ± 0.09^a^	3.52 ± 0.12^b^	127.8 ± 2.0^b^	19.7 ± 0.2^f^	108.1 ± 3.7^ab^	56.0 ± 3.9^bc^

Data expressed as mean ± SD, n = 3. Different lowercase letters within each column indicate significant differences (p-value < 0.05).

a Not determined.

**Table 6:** Gelling properties^a^ of fava bean, chickpea, and red lentil in three forms: raw (R) flour, dry-fractionated (DF) protein-enriched fractions, and wet-fractionated (WF) protein-enriched fractions.

Ingredient concentration (%w/v)	Fava bean			Chickpea			Red lentil		
Processing	R	DF	WF	R	DF	WF	R	DF	WF
2 %	xΔ	x	x	xΔ	x	x	xΔ	x	x
4 %	xΔ	x	x	xΔ	x	x	xΔ	x	x
6 %	xΔ	x	x	xΔ	x	x	xΔ	x	x
8 %	xΔ	x	x	xΔ	✓	x	xΔ	x	x
10 %	xΔ	✓	x	xΔ	✓	x	xΔ	✓	x
12 %	xΔ	✓	x	xΔ	✓	x	xΔ	✓	x
14 %	xΔ	✓	x	Δ	✓	x	xΔ	✓	x
16 %	xΔ	✓	x	Δ	✓	x	Δ	✓	x
18 %	Δ	✓	✓	Δ	✓	x	Δ	✓	x
20 %	Δ	✓	✓	x✓	✓	x✓	Δ	✓	x✓

a x - no gel; xΔ - gel < syneresis; Δ -gel ≥ syneresis; x✓ - weak gel; ✓ - firm gel

IVPDCAAS, calculated as the product of IVPD and AAS of the most limiting essential AA, provides a relatively more accurate evaluation of protein quality (Wang et al., 2023a). Dry fractionation improved the IVPDCAAS across all legume flours, increasing from 42.89 % - 45.72 % to 47.25 % - 62.43 %. In contrast, wet fractionation had mixed effects. Specifically, IVPDCAAS decreased to 43.78 % in fava bean, failing below the value of raw flour. However, wet fractionation increased the IVPDCAAS in chickpea (59.28 %) and red lentil (51.94 %). Notably, in chickpea, IVPDCAAS was higher after wet fractionation than dry fractionation, whereas in red lentil, wet fractionation yielded lower values compared to dry fractionation. While fractionation generally improved IVPDCAAS, reductions in specific essential amino acids, particularly Met + Cys, likely contributed to decreases in IVPDCAAS values.

Previous studies have investigated the nutritional quality of these legumes. Shi (2022) also found Met + Cys as limiting amino acids in fava bean flour (AAS 0.77 – 0.91) and fava bean protein isolates (AAS 0.58 – 0.62). They reported lower IVPD values (75.8 % – 78.8 %) and similar IVPDCAAS values (44.8 – 49.3) in fava bean protein isolates, but lower IVPD values (72.8 % – 73.0 %) and higher IVPDCAAS values (55.8 – 64.1) in raw flour. Li et al. (2024) also reported Met + Cys (AAS 0.7) as the most limiting amino acids in fava bean, with considerably higher IVPD values (84.5 % and 92.5 %) and IVPDCAAS values (50.7 % and 63.7 %) for dry- and wet-fractionated fava bean PF, respectively. For chickpea, Tavano et al. (2016) also identified Met + Cys (AAS 0.75) as limiting amino acids in chickpea flour, reporting a lower IVPD of 70.99 %, but a higher IVPDCAAS of 53.24 %. Goertzen et al. (2021) found that the limiting amino acid in chickpea protein isolate changed to Trp (AAS 0.83), which aligns with the findings of the present study, along with a higher IVPD (82.24 %) and IVPDCAAS (68.24 %). For red lentil, Liu et al. (2022) reported Trp (AAS 0.71) as the most limiting amino acid, with Met + Cys having only a slightly higher AAS (0.77). They observed a lower IVPD (73.6 %) but a higher IVPDCAAS (53.1 %) for red lentil flour. Thirulogasundar (2023) investigated the nutritional value of red lentil protein isolate and found that Trp (AAS 0.62) was lower than Met + Cys (AAS 0.78), with a notably higher IVPD (89 %) and IVPDCAAS (55 %). Variations in nutritional parameters across studies can be attributed to a wide diversity of factors such as varietal differences (genetic traits), extraction technology used, plant developmental stage and growth conditions (Lee et al., 2021; Trovato et al., 2021).

Soy and casein are two widely used ingredients in the food industry. The limiting amino acid in soy is Met + Cys, with and AAS, IVPD and IVPDCAAS of 0.88, 84.06 %, and 73.73 %, respectively (Nosworthy et al., 2023b). In contrast, casein has Thr as the lowest AAS (1.04), with an IVPD of 89.28 %, and IVPDCAAS of 92.85 % (Nosworthy et al., 2023a). Clearly, fava bean, chickpea, and red lentil flours exhibit lower nutritional values compared to soy and casein. To address the limitations, fractionation alone is insufficient. Alternative strategies, such as protein blending (Hertzler et al., 2020), and additional processing methods like extrusion (Nosworthy et al., 2018) and germination (Qureshi, 2023), are recommended to further enhance the nutritional quality of these legume flours.

### 3.4 Protein composition and structural properties

**Fig. 2:**
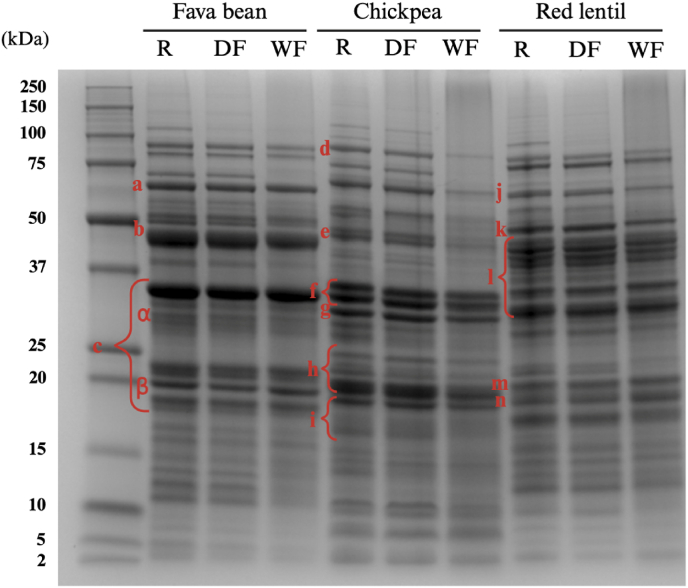
SDS-PAGE patterns of faba bean, chickpea, and red lentil in three forms: raw (R) flour, dry-fractionated (DF) protein-enriched fractions, and wet-fractionated (WF) protein-enriched fractions. Protein bands: a) Fava bean - Convicilin; b) Fava bean - Vicilin; c) Fava bean - Legumin; d) Chickpea - Lipoxygenase; e) Chickpea - Vicilin (7S Globulin); f) Chickpea - α-Legumin (11S Globulin); g) Chickpea - Vicilin (7S Globulin); h) Chickpea - β-Legumin (11S Globulin); i) Chickpea - Vicilin (7S Globulin); j) Red lentil - Convicilin; k) Red lentil - 7S Vicilin; l) Red lentil - 11S Acidic subunit; m) Red lentil – 11S Basic subunit, and n) Red lentil - γ-Vicilin.

**SDS-PAGE** was performed under reducing conditions to investigate the protein profiles of the three legumes before and after dry or wet fractionation (As shown in Fig. 2). The bands observed in the gel were identified according to the molecular weights reported by previous studies (Glusac et al., 2020; Lee et al., 2021; Żmudziński et al., 2021). The difference between raw flours and dry-fractionated PF were minor. The only notable difference observed in the gel was the disappearance of a band (∼113 kDa) in fava bean and a band (∼107 kDa) in red lentil. This finding aligned with the work of Silventoinen et al. (2021), which indicated that all protein bands were retained in protein-enriched fine fractions, compared to milled rye brans. However, they also reported that concentrated albumins (12 – 14 kDa) after dry fractionation resulted in higher band intensities, a finding that was not observed in this study. A more pronounced difference was observed in legume protein ingredients after wet fractionation. Despite the observation that most of the protein bands were retained, their intensities differed. Few differences were observed in red lentil, while several bands (∼71 kDa, ∼38 kDa, and below ∼17 kDa) appeared weaker in fava bean. Regarding chickpea, the bands at ∼18 – ∼20 kDa and ∼28 – ∼30 kDa were retained, but the intensities of other bands were noticeably lower. Kottage et al. (2024) also reported that the protein profiles of pea protein obtained by wet fractionation showed overall the same protein bands but with lower intensities compared with those produced by dry fractionation.

**Fig. 3:**
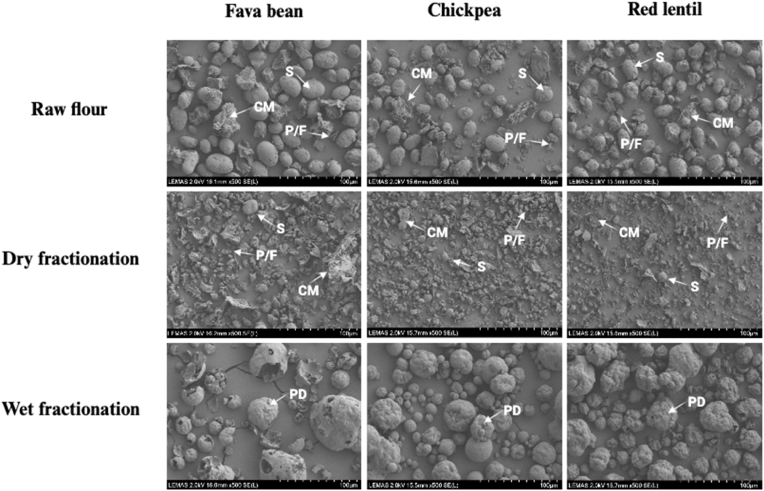
Microstructure of fava bean, chickpea, and red lentil in three forms: raw flour, dry-fractionated protein-enriched fractions, and wet-fractionated protein-enriched fractions, imaged by scanning electron microscopy (SEM). S, starch granules; CM, cellular materials; P/F, protein and/or fibre; PD, protein bodies.

**Scanning electron microscopy (SEM) micrographs** of fava bean, chickpea, and red lentil particles are presented in Fig. 3, illustrating the differences in morphological characteristics between unprocessed flours and their respective dry- and wet-fractionated PF. In raw flour, elongated and rounded starch granules (indicated by “S”) were the predominant components, while smaller, asymmetrical particles, identified as protein and/or fibre particles (“P/F”) (Schlangen et al., 2022), were also present. Additionally, irregularly shaped cellular material (denoted as “CM”) (Pelgrom et al., 2013), consisting of a combination of starch granules and protein bodies (with/without fibre), was visible. After dry fractionation, a notable reduction in starch granules was observed, indicating the effective separation of protein bodies from starch granules. Larger CM particles were found in fava bean samples, correlating with a broader particle size distribution, which was confirmed by particle size measurements (Schlangen et al., 2022; Xing et al., 2020). Following wet fractionation, a significant presence of protein bodies was observed in the SEM images. These particles exhibited a smooth and rounded morphology with noticeable shrinkage, a characteristic feature of protein isolates dried via spray-drying, attributed to water evaporation from wet droplets (Alonso-Miravalles et al., 2019). Notably, the wet-fractionated fava bean PF displayed larger particles compared to chickpea and red lentil, consistent with the particle size distribution measurements. These findings confirm the changes in particle size during the fractionation process and highlight the ability of both technologies to modify particle composition and morphology.

**Fig. 4:**
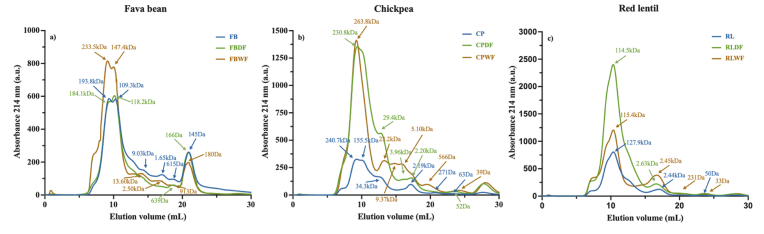
FPLC gel filtration analysis of a) fava bean; b) chickpea; and c) red lentil protein ingredients. FB, raw fava bean flour; FBDF, fava bean protein-enriched ingredient after dry fractionation; FBWF, fava bean protein-enriched ingredient after wet fractionation; CP, raw chickpea flour; CPDF, chickpea protein-enriched ingredient after dry fractionation; CPWF, chickpea protein-enriched ingredient after wet fractionation; RL, raw red lentil flour; RLDF, red lentil protein-enriched ingredient after dry fractionation, and RLWF, red lentil protein-enriched ingredient after wet fractionation.

The molecular composition of soluble fractions was determined by **fast protein liquid chromatography (FPLC)**. As shown in Fig. 4, fava bean flour exhibited three main peaks at 193.8 kDa (30.48 %), 109.3 kDa (39.16 %) and 145 Da (15.22 %), while chickpea flour displayed four predominant peaks at 240.7 kDa (31.76 %), 155.5 kDa (28.69 %), 34.3 kDa (11.91 %), and 2.19 kDa (10.93 %). Red lentil flour presented two main peaks at 127.9 kDa (85.56 %) and 2.44 kDa (11.17 %). The peak at 240.7 kDa corresponds to convicilin, while the peak at 193.8 kDa represents the legumin trimer. The peaks at 155.5 kDa and 127.9 kDa are associated with vicilin, while the 109.3 kDa peak corresponds to the legumin unit pair. The peak at 34.3 kDa represented *α-*legumin. Peaks at 2.19 kDa and 2.44 kDa are attributed to low molecular weight peptides, whereas the 145 Da peak represents free amino acids (Barać et al., 2015).

**FPLC** profiles of legumes ingredients obtained through dry fractionation are shown in Fig. 4. Fava bean and red lentil showed no apparent difference in the peaks and the ratios of major components. However, the peak corresponding to vicilin was absent in chickpea, and convicilin (230.8 kDa) was the predominant component, accounting for 68.70 % of the peak area. Wet fractionation had a relatively notable impact on the peak composition. After this processing, the M_w_ of the largest protein component in fava bean increased to 233.5 kDa from 193.8 kDa, indicating protein aggregation. Additionally, a new peak at 13.6 kDa appeared, representing 11.05 % of the peak area. This may correspond to Albumin-1 E (Warsame et al., 2020), suggesting protein dissociation. For chickpea, an increase in Mw (from 240.7 kDa to 263.8 kDa) was found in the largest protein component, also indicating protein aggregation. Notably, a peptide with a M_w_ 5.10 kDa was observed, accounting for 12.72 % of the peak area, suggesting that some protein molecules may have disassembled. Relatively less impact from wet fractionation was observed in red lentil protein. No significant difference was found in M_w_, while the ratio of high M_w_ components to low M_w_ components changed from 7.66:1 to 3.37:1, suggesting that vicilin became insoluble and/or dissociated (Scilingo et al., 2002).

**Fig. 5:**
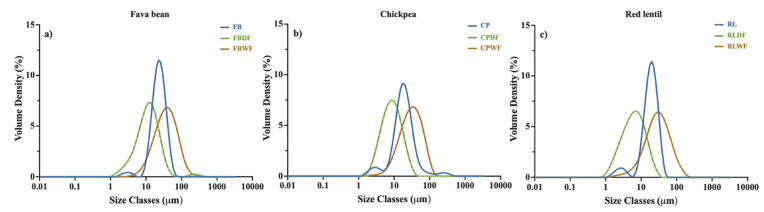
Particle size of a) fava bean; b) chickpea; and c) red lentil protein ingredients. FB, raw fava bean flour; FBDF, fava bean protein-enriched ingredient after dry fractionation; FBWF, fava bean protein-enriched ingredient after wet fractionation; CP, raw chickpea flour; CPDF, chickpea protein-enriched ingredient after dry fractionation; CPWF, chickpea protein-enriched ingredient after wet fractionation; RL, raw red lentil flour; RLDF, red lentil protein-enriched ingredient after dry fractionation, and RLWF, red lentil protein-enriched ingredient after wet fractionation.

**Particle size** is another important property of protein ingredients because it can influence functional properties and visual acceptance. A decrease in particle diameter is linked to improved WHC and foaming stability due to the increased surface area per unit volume, which can enhance the characteristics of food products (de Paiva Gouvêa et al., 2023). The volume-weighted particle size distribution for fava bean, chickpea and red lentil are presented in Fig. 5. The distribution of these three legume flours were bimodal, consisting of two parts: 1) protein bodies, ranging from 1 to 3 μm (Pernollet, 1978), and 2) starch granules, with the highest peak at ∼20 μm (Pelgrom et al., 2015b). After dry fractionation, monomodal peaks were observed in fava bean, chickpea and red lentil, with an average particle size of 15.0 ± 0.9 μm, 10.1 ± 0.1 μm and 7.7 ± 0.2 μm, respectively. The reduction in particle size of dry-fractionated PF was also observed by Rempel et al. (2019), reflecting that the protein-enriched fraction was effectively separated from starch granules.

A monomodal peak was also observed in wet-fractionated fava bean, chickpea, and red lentil protein flours, with larger particle sizes of 45.4 ± 0.6 μm, 36.4 ± 1.0 μm, and 37.9 ± 0.5 μm, respectively. The increase in particle size is likely attributed to the formation of larger aggregates, which may result from the increased viscosity associated with higher protein content (O'Donoghue et al., 2019). This could also be attributed to protein denaturation and aggregation under harsh conditions during alkaline extraction and spray-drying (Abd Rahim et al., 2023). The observed changes in particle size before and after fractionation reported in this study are generally in agreement with the findings of Li et al. (2024). However, a notable difference was observed, in their study, the highest peak for fava bean and yellow pea protein isolates obtained through wet fractionation was approximately 90 μm, whereas the corresponding peaks in this study ranged from ∼30 μm to ∼40 μm. This indicates that relatively smaller aggregates were formed, likely due to the differences in fractionation conditions and type of legumes.

The **secondary structure** of proteins in legume flours was analysed using **FTIR spectroscopy**. According to Ye et al. (2024), the spectral regions of β-sheet, random coil, α-helix, and β-turn were located at 1,610 - 1,640 cm^−1^, 1,640 - 1,650 cm^−1^,1,650 - 1,660 cm^−1^, and 1,660 - 1,700 cm^−1^, respectively. The proportions of tightly ordered structure (β-sheet + α-helix) (Ye et al., 2024) of fava bean, chickpea, and red lentil were 56.73 %, 58.95 % and 50.57 %, respectively (Shown in Table 4). Following dry and wet fractionation, the content of tightly ordered structures in fava bean and chickpea remained unchanged. However, a significant increase was observed in red lentil, with tightly ordered structures increasing to 59.65 % and 59.55 % after dry and wet fractionation. For random coil structure, a significant decrease was observed in chickpea after dry fractionation, from 30.02 % to 22.91 %. Conversely, red lentil exhibited a significant increase in random coil content after wet fractionation, from 20.57 % to 30.56 %. The β-turn content in chickpea increased significantly after dry fractionation, rising from 11.01 % to 18.46 %. In contrast, the β-turn in red lentil showed a remarkable reduction, decreased from 28.25 % to 17.71 % after fractionation and further decreasing to 9.88 % after wet fractionation. Apart from the aforementioned samples, the fractionation process did not exert a significant effect on random coil and β-turn structures in other legumes.

The comparison of secondary structure of legume flours with previous studies was challenging due to differences in legume cultivars, growth location, absence of standardized protocols for ingredients preparation, and differences in equipment used for identifying secondary structures. Jeganathan et al. (2024) reported similar α-helix (23.24 %) and β-turn (14.57 %) proportions in fava bean. However, a much higher β-sheet proportions, accounted for 57.16 %. In this study, a significant higher proportion of random coil (29.57 – 34.80 %) was found. This finding was supported by Liu et al. (2017), who predicted that random coil in storage protein in fava beans ranged from 23.28 % to 38.43 %. In addition, Withana-Gamage et al. (2011) measured the secondary structure of Kabuli and Desi chickpea protein, reporting β-sheet (32.5 % – 40.4 %), random coil (16.3 % – 19.2 %), α-helix (25.6 % – 32.7 %), and β-turn (13.8 – 18.9 %) contents. β-sheet and α-helix contents were similar to those of the present study but with lower random coil proportions and higher β-turn content. The higher random coil content observed in this study may be attributed to manufacturing techniques that led to slight protein unfolding compared to lab-scale extractions (Buscajoni et al., 2022; Fitzkee and Rose, 2004). The impact of fractionation methods on secondary structure is complex and protein-dependent, consistent with the findings reported by De Angelis et al. (2024) and Li et al. (2024).

**Surface hydrophobicity (H_0_)** of protein reflects the distribution of hydrophobic residues on the surface and is closely associated with its interfacial and functional properties (Tang et al., 2021). Among unprocessed flours, fava bean had the highest H_0_ (44,243), followed by red lentil (32,492). Dry fractionation had varied impact on the PF, specifically H_0_ value was reduced in faba bean (-34.32 %), increased in chickpea (+26.14), and remained unchanged in red lentil. Wet fractionation resulted in a pronounced increase in H_0_ among all samples. Wet-fractionated chickpea and red lentil showed similar H_0_ values (92,915 and 99,381), while H_0_ for fava bean was relatively lower (62,739).

Non-proteinaceous compounds, such as starch have lower surface hydrophobicity compared to proteins (Scott and Awika, 2023). They may also hinder or interfere with hydrophobic domains of proteins (Yang et al., 2023), thereby reducing the surface hydrophobicity of proteins. As a result, protein-enriched ingredients have attracted significantly more interest in the literature than raw flours. For instance, Karaca et al. (2011) reported that the surface hydrophobicity of fava bean protein isolates was 55.23 H_0_, which was lower than that of red lentil protein (64.67 H_0_) and chickpea (80.36 H_0_), consistent with the findings reported in the present study.

In this study, the impact of dry fractionation on H_0_ largely depended on the type of legume. This can be attributed to the fact that dry fractionation does not extensively disrupt protein structures, allowing non-proteinaceous compounds to remain in place and continue blocking hydrophobic regions. Consequently, the effect of surface hydrophobicity is largely influenced by the extent of removal of these obstructing compounds. In contrast, wet fractionation has been well-documented to increase H_0_. Vogelsang-O’Dwyer et al. (2020) observed a marked increase in H_0_ for fava bean protein isolated through isoelectric precipitation (2,183), compared to PF obtained thought dry fractionation (1,208). Similarly, Kottage et al. (2024) reported that H_0_ of wet-fractionated pea PF was 2,928.41, significantly higher than that of dry-fractionated PF (1,526.92). The increase in H_0_ can be attributed to protein denaturation caused by changes in pH and the high temperatures associated with spray-drying. These conditions induce protein unfolding, exposing previously buried hydrophobic regions and consequently enhancing surface hydrophobicity (Hall and Moraru, 2021).

### 3.5 Techno-functional properties

Techno-functional properties of proteins, including water holding capacity, oil holding capacity, foaming properties, emulsifying properties, protein solubility, gelation, particle size, and zeta-potential, are known to determine the behaviour of proteins in food processing technologies and play an important role in new food product development. In this study, the techno-functional properties of fava bean, chickpea, and red lentil were measured, and the impact of dry fractionation and wet fractionation is presented.

**Water holding capacity (WHC)** reflects the ability of protein flour to absorb water without dissolving. Since most food applications of protein flours largely depend on the interaction between protein and water, this property plays a crucial role in determining the body and texture of food products, as well as influencing thickening, viscosity, and other sensory properties (Sreerama et al., 2012). As shown in Table 5, chickpea flour exhibited the highest WHC at 1.71 g/g, followed by red lentil (1.26 g/g) and fava bean (1.08 g/g). Makri et al. (2005) reported a lower WHC for chickpea four at 1.19 g/g, whereas Du et al., (2014) documented a WHC range of 1.33 g/g to 1.47 g/g among six chickpea cultivars. For red lentil flour, slightly higher WHC was reported by Marchini et al. (2021) at 1.68 g/g while (Kaur and Sandhu, 2010) observed a WHC range of 1.5 g/g to 1.7 g/g among four red lentil cultivars. Moreover, Olakanmi et al. (2024) reported a relatively comparable WHC of 0.96 g/g for fava bean flour, whereas Oluwajuyitan and Aluko (2024) presented a higher WHC of 1.65 g/g.

No significant difference in WHC was found in chickpea before and after dry fractionation. However, WHC of fava bean and red lentil improved to 1.37 g/g and 2.03 g/g, respectively, after dry fractionation. Comparatively, these results differed from the findings of Oluwajuyitan and Aluko (2024), who reported that WHC of fava bean decreased to 0.62–0.71 g/g from 1.65 g/g. Moreover, De Angelis et al. (2021a) also observed a decrease in WHC for red lentil (-0.04 g/g), yellow lentil(-0.15 g/g), green pea (-0.23 g/g) and kabuli chickpea (-0.46 g/g). Schlangen et al. (2022) observed a similar increasing trend in WHC for mung bean and cowpea, but a decrease in yellow pea. Dry fractionation resulted in an increased protein content and a reduction in starch content. The presence of polar and charged amino acid residues within the protein chains enhances water retention through hydrogen bonding and electrostatic interactions (Raschke, 2006). In contrast, starch which contains fewer polar groups forms weaker interactions with water (Scott and Awika, 2023). Additionally, the increase in TDF, ranging from +0.88 g/100 g–4.52 g/100 g, has been positively correlated with WHC due to its porous network (Liu et al., 2025). Therefore, the overall WHC is likely the result of the combined effects of these components. Variations in the contribution of residual fibre to water retention may explain the observed difference in WHC among legume PF after dry fractionation. A continuous increase in WHC was found after wet fractionation in all three legumes (increased to 2.21–2.83 g/g). This finding aligned with Hopf et al. (2024), who reported that chickpea (+2.8 g/g), fava bean (+3.5 g/g), and mung bean (+4.2 g/g) exhibited higher WHC in commercial products produced via wet fractionation compared to dry fractionation. Additionally, the average WHC (2.9 8 g/g) of wet-fractionated commercial plant protein ingredients (oat, chickpea, lentil, pea, hemp, soy and wheat gluten) was higher than those obtained (chickpea, lentil, pea, mung bean, grass pea, and fava bean) by dry-fractionation (0.77 g/g) (De Angelis et al., 2024). This may be due to protein denaturation during the wet fractionation process, since denatured proteins have a higher WHC compared to its native form (Bühler et al., 2020).

**Oil holding capacity (OHC)** indicates the ability of a protein ingredient to entrap oil (fat). Since oil (fat) retains flavours and enhances mouthfeel, OHC is considered as an important functional property in food applications (Khattab and Arntfield, 2009). Fava bean and chickpea flour showed similar OHC, which were 3.45 g/g and 3.40 g/g, respectively. Olakanmi et al. (2024) reported similar OHC for fava bean flour (3.66 – 3.86 g/g). However, a lower OHC for fava bean (1.65 g/g) was found by Oluwajuyitan and Aluko (2024). Much lower OHC for chickpea has been reported in previous studies, for example, 0.81 g/g (Jagannadham et al., 2014) and 1.05 – 1.24 g/g (Kaur and Singh, 2005). Similarly, OHC of red lentil was reported at 1.32–1.39 g/g (Bourré et al., 2019) and 0.92 – 1.13 g/g (Kaur and Sandhu, 2010), which are much lower than the value reported in this study (3.17 g/g).

No significant difference was found in fava bean, while dry fractionation significantly improved OHC in chickpea (+0.58 g/g) and red lentil (+0.71 g/g). A similar trend was reported by Oluwajuyitan and Aluko (2024), where OHC in fava bean increased from 0.69 g/g to 0.99 – 1.09 g/g after dry fractionation. Fenn et al. (2022) also observed that dry fractionation improved OHC in yellow pea from 0.54 to 0.57 g/g to 0.93–0.97 g/g. Similarly, do Carmo et al. (2020) reported that the protein-rich fraction of pea (1.12 g/g vs. 0.77 g/g) and chickpea (1.15 g/g vs. 0.82 g/g) had higher OHC compared to their starch-rich fractions, indicating that fractions with higher protein content tend to exhibit improved OHC. However, a further increase in protein content induced by wet fractionation failed to enhance the OHC, even in fava bean (2.78 g/g) and chickpea (3.16 g/g), where the OHC was lower than the one reported for the unprocessed flour. This finding contrasted with previous reports. For example, Hopf et al. (2024) reported higher OHC in chickpea (1.4 g/g vs. 1.0 g/g), fava bean (1.5 g/g vs. 1.1 g/g), and mung bean (1.7 g/g vs. 1.0 g/g) produced by wet fractionation compared to dry fractionation. Ma et al. (2022) reviewed OHC of five flours, fourteen protein concentrates and twenty-nine protein isolates, and suggested a positive correlation between OHC and protein content, which generally aligned with the data from dry fractionation, but contradicted findings related to wet fractionation. The discrepancy was attributed to the exposure of the hydrophobic core of proteins after denaturation, as indicated by the increase in H_0_ values of legume flours from 26,001 to 44,243 to 62,739 – 99,831 after wet fractionation. This change reduced their oil-binding capacity (Zhu et al., 2017).

**Foaming properties** involve protein flour creating air bubbles at the air-water interface and maintaining these bubbles in suspension. Foaming capacity (FC) and foaming stability (FS) are two common parameters used to evaluate this property. The former indicates the volume of foam produced, while the latter represents the ability to retain the bubbles. FC of chickpea was 50.9 %, which was much lower than that of fava bean (115.6 %) and red lentil (113.3 %). However, FS of chickpea showed the highest percentage (93.1 %), compared to fava bean (71.2 %) and red lentil (71.0 %). Dry fractionation led to an increase in FC of chickpea (+25.5 %) but resulted in a decrease in fava bean (-30.0 %) and red lentil (-20.6 %). In terms of FS, dry fractionation had a slight effect, ranging from -9.7 % to +4.5 %. Smaller air bubbles were visually observed after dry fractionation, likely due to the removal of starch and available carbohydrates, which resulted in a reduction in viscosity (Stewart, 1995). In addition, although dry fractionation increased the overall protein content, the protein solubility decreased, for example, from 60.1 % to 30.1 % in red lentil, suggesting that soluble protein fraction did not increase substantially thereby limiting improvements in FC (Moll et al., 2022). Moreover, surface hydrophobicity of fava bean proteins decreased from 44,243 H_0_ to 29,508 H_0_, contributing to a reduction in FC (Zhu et al., 2017). These factors led to a decrease in FC. The contrasting trend observed in chickpea may be attributed to an increase in saponins, from 807.4 to 1,616.2 g/100 g, as saponins are known to act as natural foaming agents (Timilsena et al., 2023). Regarding the minor changes observed in FS, this is likely because dry fractionation has a limited impact on the overall protein profile (e.g., protein fractions observed by SDS-PAGE), which would be expected to have only a limited influence on FS.

Regarding wet fractionation, compared to unprocessed flour, an increase in FC was observed in Fava bean (+23.5 %) and red lentil (+14.5 %), However, the bubbles generated by wet-fractionated chickpea PF rapidly collapsed, resulting in a FC value of zero, thereby preventing the determination of FS. A significant decrease in FS was observed in fava bean and red lentil, with values sharply reduced to 49.2 % and 19.7 %, respectively. The observed increase in FC can be attributed to an enhanced surface hydrophobicity, which improves the ability of proteins to adsorb at the air-water interface (Zhu et al., 2017). Additionally, the presence of aggregated proteins reduces surface tension and, when combined with non-aggregated proteins, facilitate the formation of larger and more stable air bubbles (Hu et al., 2019; Rullier et al., 2008). However, in this study, the FS was decreased likely due to the separation of albumin from globulin during the wet fractionation extraction steps, resulting in weaker interfacial layers around air bubbles (Möller et al., 2022). Furthermore, the substantial removal of starch and carbohydrate led to a decrease in the viscosity of the continuous phase, further contributing to the reduction in FS (Silventoinen et al., 2021).

Numerous studies have evaluated FC and FS in these legume flours. For instance, Olakanmi et al. (2024) reported that FC and FS of fava bean flour were ∼70 % and ∼90 %, respectively. In contract, Oluwajuyitan and Aluko (2024) claimed a much lower FC (∼33 %), but FS still at ∼90 %. Additionally, Raikos et al. (2014) reported a comparable FS (∼70 %), but a lower FC (∼75 %) in fava bean flour. Regarding chickpea, Sreerama et al. (2012) observed a similar FC (46.3 %) in chickpea flour, but a relatively low FS (39.2 %). Conversely, Jagannadham et al. (2014) reported a similar FS (96.91 %), but a much lower FC (29.27 %). Compared to this study, significant differences were observed in the FC and FS of red lentil flours which were 57.1 % and 43.2 %, respectively, as reported by Badia-Olmos et al. (2023).

Unlike the findings in this study, Fenn et al. (2022) observed an increase in FC for four yellow pea cultivars, with their FC increasing from 212 - 246 % to 245 - 305 % after dry fractionation. In contrast, FS exhibited no statistically significant change. Oluwajuyitan and Aluko (2024) observed an overall negative impact of dry fractionation on FC and a slight increase in FS, which aligned with this study. The comparison between dry fractionation and wet fractionation has also been evaluated by previous studies, for example, dry-fractionated chickpea PF exhibited a lower FC (14.7 %) compared with wet fractionated PF (41.7 %) (Hopf et al., 2024). Fava bean flour showed the opposite trend, with FC decreasing from 67.1 % to 39.2 %. FS decreased in chickpea flour from 95 % to 74 %, while no significant changes in FS were detected for fava bean flour (Hopf et al., 2024). Additionally, De Angelis et al. (2024) compared plant-based ingredients obtained though dry fractionation and wet fractionation, they reported that the average FC value was higher in dry fractionated PF (112.03 % vs. 182.92 %). However, FS was lower in wet-fractionated samples compared to dry-fractionated ones (39.91 % vs. 74.74 %). The substantial variation observed across studies may be attributed to several factors, including differences in homogenization speed, foaming duration, protein concentration, and the technology used to produce the protein ingredients.

**Emulsifying property** refers to the ability of proteins to act as emulsifiers, forming a layer around oil droplets that are dispersed in the water phase to prevent phase separation. In this study, this property was evaluated by emulsifying capacity (EC) and emulsifying stability index (ESI). EC refers to the interfacial area stabilized per unit weight of protein, while ESI indicates the ability of the protein to retain the emulsion structure (Thaiphanit et al., 2016). Red lentil flour presented the highest EAI at 75.2 m^2^/g, followed by chickpea flour (67.9 m^2^/g). Chickpea flour showed the best ESI (65.4 min), while only a small difference was observed between fava bean flour (30.9 min) and red lentil flour (33.6 min). Dry fractionation led to an increase in EAI, ranging from +11.4 m^2^/g to +33.9 m^2^/g. An increase in ESI was also found in chickpea (+106.9 min) and red lentil (+24.0 min) after dry fractionation, but no significant change was observed in fava bean. The significant increase in EAI and ESI observed in dry-fractionated chickpea and red lentil PFs can be primarily attributed to their elevated dietary fibre content, which can act as natural emulsifiers, helping to prevent droplet aggregation and gravitational separation (Zhang et al., 2019). Furthermore, the smaller particle size of dry-fractionated PF contributed to the enhancement of both EAI and ESI (Zhou et al., 2025). Regarding fava bean, the reduction in surface hydrophobicity (-15,185 H_0_) limited improvements in emulsifying properties (Dong et al., 2023), which may explain the slight increase in EAI and the lack of significant change in ESI following dry fractionation.

Compared to dry fractionation, wet fractionation further increased the EAI in fava bean (+29.3 m^2^/g), while no significant change was observed in chickpea and red lentil. However, wet fractionation had a negative impact on the ESI of fava bean (-9.3 min) and chickpea (-13.2 min), with values even lower than those of the unprocessed flours, while no significant change was observed in red lentil. The increase in surface hydrophobicity following wet fractionation contributed positively to the emulsifying properties of PF by strengthening protein-oil interactions. However, the formation of denatured and aggregated proteins during wet processing can result in inhomogeneous interfacial layers, thereby reducing ESI (De Angelis et al., 2024). Furthermore, the removal of carbohydrates may also contribute to reduced ESI, as carbohydrate help stabilize emulsions through steric hindrance and electrostatic repulsion (Wang et al., 2021).

Comparison of emulsifying properties with previous studies on legume flours were challenging, as most studies measured the EAI and ESI of protein concentrates/isolates rather than flours. For example, Shi and Nickerson (2022) reported that the EAI and ESI of protein concentrates from three fava bean cultivars were ∼12 m^2^/g - ∼31 m^2^/g and ∼12min – ∼25min, respectively. do Carmo et al. (2020) reported that EAI and ESI for fava bean protein concentrate was 11.80 m^2^/g and 14.24 min, respectively. Karaca et al. (2011) found that EAI of chickpea, fava bean and lentil protein concentrate was 47.9, 44.3 and 44.5 m^2^/g, respectively, with corresponding ESI values of 82.9 min, 69.4 min, and 86.8 min. Lee et al. (2021) reported much lower EAI and ESI values for red lentil protein concentrate with ∼29 m^2^/g and ∼15 min, respectively. These data largely disagreed with the results of this study. Regarding the comparison between dry fractionation and wet fractionation. There were obvious discrepancies among studies, which were not only due to differences in sample types but also due to variations in experimental conditions, such as volume, speed, oil types, and sample dispersion (Lee et al., 2021). EAI and ESI reflect the emulsifying properties of the whole system, rather than the properties of the legume ingredients alone.

**Fig. 6:**
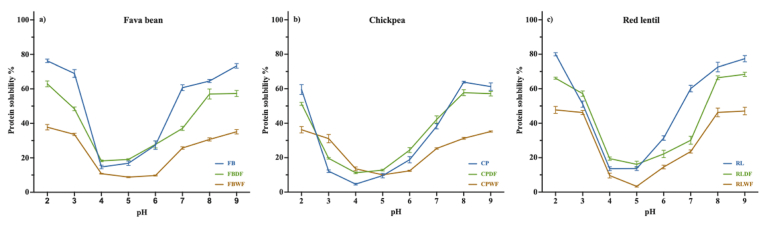
Protein solubility (%) of a) fava bean, b) chickpea, and c) red lentil protein ingredients. FB, raw fava bean flour; FBDF, fava bean protein-enriched ingredient after dry fractionation; FBWF, fava bean protein-enriched ingredient after wet fractionation; CP, raw chickpea flour; CPDF, chickpea protein-enriched ingredient after dry fractionation; CPWF, chickpea protein-enriched ingredient after wet fractionation; RL, raw red lentil flour; RLDF, red lentil protein-enriched ingredient after dry fractionation, and RLWF, red lentil protein-enriched ingredient after wet fractionation. Data expressed as mean ± SD, n = 3.

**Protein solubility (PS)** measures the amount of protein solubilized in water, and it is strongly affected by pH. **PS** is closely associated with other functional properties, such as emulsification and gelation, and directly determines the utilisation of protein ingredients in food formulation. Fig. 6 illustrates the PS of legume flours across a pH range of 2–9, showing a typical U-shaped curve. Solubility was highest under highly acidic (pH 2) and/or alkaline (pH 9) conditions, while the lowest solubility was observed at pH 4 and/or pH 5. This is attributed to the isoelectric points of proteins in these legume flours, which were between pH 4 and pH 5. At isoelectric points, the net surface charge of a protein is zero, leading to protein aggregation and precipitation (Lee et al., 2021). Dry fractionation resulted in a slight increase in PS near the isoelectric point, however, PS significantly decreased under most other pH conditions. PS for chickpea flour at pH 3 (31.08 %) and pH 4 (13.57 %) was highest after wet fractionation, compared to raw flour (15.14 % and 4.66 %) and dry-fractionated PF (19.65 % and 11.24 %). Despite an increase in PS was found at both specific pH conditions, a huge decrease was found under other conditions. For example, maximum PS within the pH range of 2–9 decreased from 63.68 to 80.01 % (unprocessed flour) to 47.72 – 35.18 % (wet-fractionated PF).

Previous studies have investigated the impact of dry fractionation on protein solubility. Silventoinen et al. (2021) reported a decrease in protein solubility of rye bran from 42.2 % to 34.9 % under native pH after dry fractionation. This finding was consistent with the results of the present study. However, the same study also found that protein solubility of wheat bran did notchange after processing (43.9 % vs. 45.1 %, p-value >0.05). In contrast, Oluwajuyitan and Aluko (2024) reported a significant increase in protein solubility of dry-fractionated fava bean PF. The large disagreement in the literature suggests that the impact of dry fractionation on protein solubility is highly dependent on protein ingredients, protein fraction ratios (albumin/globulins/glutenins/prolamins) and processing parameters.

Extensive evidence indicated that wet fractionation negatively impacted protein solubility. Kottage et al. (2024) reported that the maximum PS of dry-fractionated pea PF was 53.78 %, significantly higher than that of wet-fractionated pea protein (17.21 %). Additionally, Hopf et al. (2024) observed higher PS of chickpea (79.6 % vs 45.7 %), fava bean (79.7 % vs 8.7 %), and mung bean (79.5 % vs 5.9 %) when compared to wet fractionated counterparts at neutral pH. Similarly, Vogelsang-O’Dwyer et al. (2020) observed that PS of dry-fractionated fava bean PF was higher than that of protein isolated by isoelectric precipitation. They suggested that the reduction in PS after wet fractionation was due to protein denaturation resulting from the extraction and spray-drying steps. Overall, dry fractionation led to moderate protein aggregation, resulting in a slight decrease in PS. In contrast, the extensive protein aggregation and denaturation during the wet fractionation led to a more pronounced decrease in PS (Kottage et al., 2024; Möller et al., 2022).

**Gelation** is associated with the capacity of denatured proteins to form a three-dimensional network, which is important for developing the textural and rheological characteristics of food products. The gel formed by raw flours appeared as ‘viscous paste’, which was characteristic of starch gelation rather than protein gelation (BeMiller, 2011). This physical process involved the swelling and breaking of starch granules, which form hydrogen bonds with water and form a thick solution (Schmiele et al., 2019). As shown in Table 6, at a 20 % concentration, only a weak protein gel was observed in unprocessed chickpea flour, indicating starch play a dominant role in gelation. The proteins entrapped within the starch matrix appear to play a minor role in gel formation (Scott and Awika, 2023).

The least gelling concentration (10.13039/501100003360LGC) refers to the minimum concentration of protein required to form a self-supporting gel and serves as an indicator of the gelation behaviour of proteins. A lower LGC indicates that less protein is needed for forming a gel, contributing to an increased sustainability and affordability (Schlangen et al., 2022). The LGC of the three dry-fractionated protein-enriched legume PF reported in this work ranged from 8 % - 10 %. Although gelation properties were influenced by protein content, cultivar types, pH and preparation methods, similar LGC ranges have been reported in the literature. For examples, LGC values of 8 % - 10 % for fava bean (Kamani et al., 2024) and 10 % lentil protein (Jo et al., 2020) were reported. Similarly, Boye et al. (2010a) also reported LGC of red lentil and chickpea between 10 - 12 % and 10 – 14 %, respectively. Dry fractionation increases protein content while selectively removing the starch fraction. The residual starch contributes positively by occupying space, absorbing water, and participating in protein-polysaccharide interactions, thereby facilitating the formation of a stronger gel network (Guo et al., 2024).

However, after wet fractionation, the gelation properties were adversely affected. In this study, the LGC of fava bean increased to 18 %, whereas chickpea and red lentil formed only weak gels even at a 20 % concentration. Kaur and Singh (2007) similarly reported higher LGC values for protein isolates obtained from six chickpea cultivars using alkaline extraction, with values ranging from 14 % to 20 %, compared with flour (10 – 14 %). They suggested that non-protein components, such as polysaccharides, also contribute to gelation. Moreover, the harsh conditions during wet fractionation resulted in an increased presence of insoluble protein particles, which negatively affected gelation properties (Tiong et al., 2025). In contrast, wet fractionation effectively removes most of the starch and significantly increases the protein content. However, the extracted proteins were prone to denaturation and aggregation. These structural alterations not only reduced the availability of soluble proteins essential for gel formation and hindered the formation of new intermolecular interactions during heating, which are essential for maintaining a stable gel network (Zhang et al., 2023b). As a result, gelation properties are weakened.

**Fig. 7:**
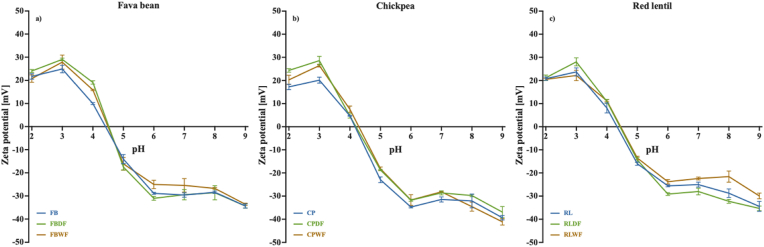
Zeta potential [mV] of a) fava bean, b) chickpea, and c) red lentil protein ingredients. FB, raw fava bean flour; FBDF, fava bean protein-enriched ingredient after dry fractionation; FBWF, fava bean protein-enriched ingredient after wet fractionation; CP, raw chickpea flour; CPDF, chickpea protein-enriched ingredient after dry fractionation; CPWF, chickpea protein-enriched ingredient after wet fractionation; RL, raw red lentil flour; RLDF, red lentil protein-enriched ingredient after dry fractionation, and RLWF, red lentil protein-enriched ingredient after wet fractionation. Data expressed as mean ± SD, n = 3.

**Zeta-potential** describes the surface changes on proteins. As shown in Fig. 7, zeta-potential changed from the maximum to minimum value when the pH increased from 2 to 9. The deprotonation of carboxyl and amino groups was responsible for this phenomenon (Tang and Sun, 2011). The maximum positive zeta-potential values for fava bean, chickpea, and red lentil were +24.9 mV, +20.2 mV, and +23.7 mV, respectively, all observed at pH 3. Subsequently, zeta-potential decreased to - 34.3 mV, - 39.4 mV, - 34.5 mV at pH 9. The isoelectric points (IP) for fava bean, chickpea, and red lentil were observed at pH ∼4.4, ∼4.2 and ∼4.3, respectively. Previous papers reported similar IP for fava bean (4.4 – 4.5) (Rahma, 1988), chickpea (∼4.5)(Adal, 2022) and red lentil (∼4.5) (Alonso-Miravalles et al., 2019). The slight differences may be attributed to minor changes in amino acid profiles and ratio of storage protein (albumin/globulin) resulting from variations between cultivars and growth conditions.

Compared with protein solubility, the impact of fractionation on zeta-potential was minimal, with a maximum pH change of ±0.2 in the isoelectric point. This finding aligned with Vogelsang-O’Dwyer et al. (2020), who also observed slight changes in the zeta-potential curve between dry-fractionated faba bean PF and wet-extracted fava bean PF. This indicates that differences in protein solubility were not related to changes in zeta-potential, but were primarily influenced by processing conditions that can lead to protein denaturation (Jiang et al., 2016) or hindered dehydration (Crowley et al., 2015).

### 3.6 Pearson correlation coefficient analysis and principal component analysis

Pearson correlation analysis was performed to assess the impact of both dry and wet fractionation on the measured variables (**Appendix A.2.**). Following dry fractionation, protein content (0.9497), condensed tannins (0.9444), and saponins (0.8223) exhibited strong positive correlation. In contrast, total starch (-0.9784), available carbohydrates (-0.8442), Trp (-0.8371), D_50_ (-0.8662), LGC (-0.9879), and protein solubility at pH 7 (-0.9445) showed strong negative correlations.

In the case of wet fractionation, several components demonstrated significant positive correlations, including protein content (0.9614), condensed tannins (0.9133), saponins (0.9341), trypsin inhibitors (0.9157), Ser (0.8723), Ile (0.8695), Leu (0.9368), and PER_1_ (0.9392), PER_2_ (0.9497), PER_3_ (0.9015), D_50_ (0.9215), surface hydrophobicity (0.8973), WHC (0.9008), and EAI (0.9060). On the other hand, wet fractionation was negatively correlated with total starch (-0.9938), available carbohydrates (-0.8622), TDF (-0.9046), Gly (-0.8623), moisture content (-0.8814), LGC (-0.8165), protein solubility at pH 7 (-0.9765), and FS (-0.8653).

**Fig. 8:**
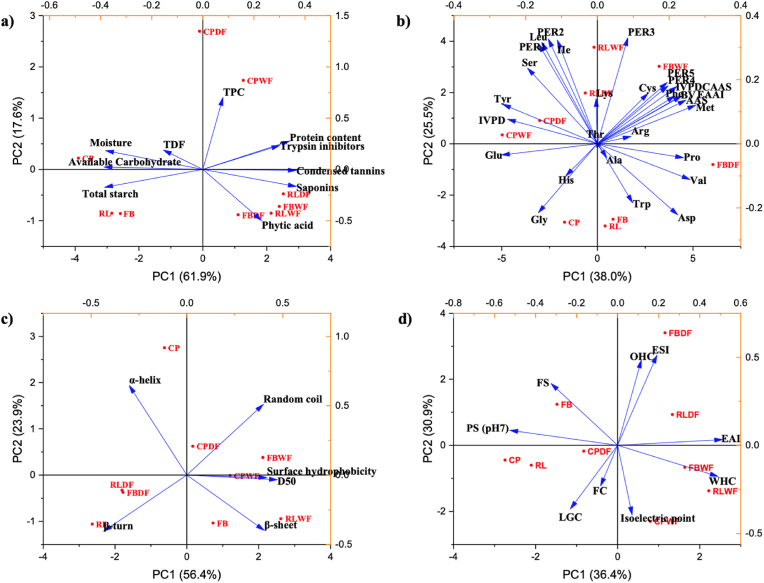
Principal component analysis (PCA) plots of a) ingredient composition and antinutritional factors; b) nutritional values; c) structural features; and d) techno-functional properties for FB, raw fava bean flour; FBDF, fava bean protein-enriched ingredient after dry fractionation; FBWF, fava bean protein-enriched ingredient after wet fractionation; CP, raw chickpea flour; CPDF, chickpea protein-enriched ingredient after dry fractionation; CPWF, chickpea protein-enriched ingredient after wet fractionation; RL, raw red lentil flour; RLDF, red lentil protein-enriched ingredient after dry fractionation, and RLWF, red lentil protein-enriched ingredient after wet fractionation. TDF, total dietary fibre; TPC, total polyphenol content; Gly, glycine; Lys, lysine; Glu, glutamine; Ser, serine; Ala, alanine; Leu, leucine; Met, methionine; Phe, phenylalanine; Trp, tryptophan; Pro, proline; Val, valine; Ile, isoleucine; Cys, cysteine; Tyr, tyrosine; His, histidine; Arg, arginine; Asn, asparagine; Asp, aspartic acid; Thr, threonine; EAAI, essential amino acid index; AAS, amino acid score; BV, predicted biological value; PER1-5, Protein efficiency ratio; IVPD, *In vitro* protein digestibility; IVPDCAAS, *In vitro* protein digestibility-corrected amino acid score; D50, average particle size; WHC, water holding capacity; OHC, oil holding capacity; FC, foaming capacity; FS, foaming stability; EAI. emulsifying activity index; ESI, emulsifying stability index; PS (pH7), protein solubility at pH 7; and LGC, least gelation concentration.

To evaluate the characteristics of legume ingredients developed through dry and wet fractionation, four PCAs were conducted, focusing on 1) ingredient composition and antinutritional factors; 2) nutritional values; 3) structural features; and 4) techno-functional properties (Fig. 8). The first two principal components (PC1 and PC2) explained 79.5 %, 63.5 %, 80.3 %, and 67.3 % of the total variance for each dataset, respectively.

In terms of ingredient composition and antinutritional factors, and nutritional values, raw red lentil and fava bean flours exhibited similarities, indicated by their close clustering. However, they appeared in a different quadrant than chickpea flour, suggesting differences. For structural features, red lentil, fava bean, and chickpea flour located separately, indicating substantial structural variation among them. Chickpea and red lentil flour clustered in the same quadrant when techno-functional properties were analysed, while fava bean flour was positioned separately, indicating a significant difference in functionality.

Wet fractionation exhibited a pronounced influence across all legumes studied, as evidenced by the shifts in different quadrant compared to the corresponding unfractionated flours. Dry fractionation also exerted significant impact, but these were more selective. Specifically, dry fractionation affected 1) fava bean and red lentil (in ingredient composition and antinutritional factors and techno-functional properties); 2) chickpea and red lentil (in nutritional values); and 3) fava bean and chickpea (in structural features). Notably, red lentil and fava bean were in the same quadrant after dry fractionation and wet fractionation, suggesting that the fractionation method did not significantly affect their composition and level of antinutritional factors. Similar patterns were observed between chickpea and red lentil when analysing their nutritional values.

Taken together, PCA underscore that influence of wet fractionation on legume flours is more pronounced, whereas the variation induced by dry fractionation appears to be legume-specific and dependent on the characteristics analysed.

## 4 Conclusion

As expected, this study demonstrates that fractionation processing more effectively concentrates protein in fava bean and red lentil than in chickpea. Although antinutritional factors accumulated, particularly after dry fractionation, IVPD was found to be maintained or even improved. This enhancement is attributed to the disruption of the starch-protein matrix (as revealed by SEM analysis), which increased protease accessibility and mitigated the inhibitory effects of antinutritional compounds. Dry fractionation was found to negatively impact Trp levels, whereas wet fractionation led to improved levels of Ile, Ser, and Leu, across all legume samples. Dry fractionation also conferred superior functional properties for legumes, minimal structural modification with retention of the complete protein profile. Whereas, wet-fractionated protein isolates exhibited enhanced emulsifying and foaming capacities, albeit with limited stability. Overall, dry fractionation is a promising method for producing functional ingredients, such as those used for producing texturized vegetal proteins. While wet fractionation remains valuable for generating protein isolates with enhanced essential amino acid content and higher protein purity.

## CRediT authorship contribution statement

**Ruixian Han:** Writing – review & editing, Visualization, Validation, Methodology, Investigation, Formal analysis, Data curation. **Yan Wang:** Validation, Methodology, Investigation, Data curation. **Zhanming Yang:** Validation, Methodology, Investigation, Data curation. **Stuart Micklethwaite:** Methodology, Data curation. **Martin Mondor:** Writing – review & editing, Validation, Supervision. **Evi Paximada:** Writing – review & editing, Validation, Supervision, Investigation. **Alan Javier Hernández-Álvarez:** Writing – review & editing, Visualization, Validation, Supervision, Resources, Project administration, Methodology, Investigation, Data curation, Formal analysis, Conceptualization.

## Declaration of competing interest

The authors declare that they have no known competing financial interests or personal relationships that could have appeared to influence the work reported in this paper.

## Data Availability

No data was used for the research described in the article.

## References

[bib1] Abd Rahim F.N., Ibadullah W.Z.W., Saari N., Brishti F.H., Mustapha N.A., Ahmad N., Arulrajah B. (2023). The effect of alkaline extraction and drying techniques on the physicochemical, structural properties and functionality of rice bran protein concentrates. Int. J. Biol. Macromol..

[bib2] Adal E. (2022). Complex coacervation of chickpea protein isolate and pectin: effect of biopolymer ratio and pH. Gıda.

[bib3] Adamidou S., Nengas I., Grigorakis K., Nikolopoulou D., Jauncey K. (2011). Chemical composition and antinutritional factors of field peas (pisum sativum), chickpeas (cicer arietinum), and faba beans (Vicia faba) as affected by extrusion preconditioning and drying temperatures. Cereal Chem..

[bib4] Adhikari S., Schop M., de Boer I.J., Huppertz T. (2022). Protein quality in perspective: a review of protein quality metrics and their applications. Nutrients.

[bib5] Affrifah N.S., Uebersax M.A., Amin S. (2023). Nutritional significance, value‐added applications, and consumer perceptions of food legumes: a review. Legume Science.

[bib6] Ajay A., Gaur S.S., Shams R., Dash K.K., Mukarram S.A., Kovács B. (2024). Chickpeas and gut microbiome: functional food implications for health. Heliyon.

[bib7] Alonso R., Aguirre A., Marzo F. (2000). Effects of extrusion and traditional processing methods on antinutrients and in vitro digestibility of protein and starch in faba and kidney beans. Food Chem..

[bib8] Alonso-Miravalles L., Jeske S., Bez J., Detzel A., Busch M., Krueger M., Wriessnegger C.L., O'Mahony J.A., Zannini E., Arendt E.K. (2019). Membrane filtration and isoelectric precipitation technological approaches for the preparation of novel, functional and sustainable protein isolate from lentils. European Food Research and Technology.

[bib9] Amin A., Petersen I.L., Malmberg C., Orlien V. (2022). Perspective on the effect of protein extraction method on the antinutritional factor (ANF) content in seeds. ACS Food Sci. Technol..

[bib10] AOAC (1990). Official methods of analysis. Assoc Anal Chem.

[bib11] Asen N.D., Badamasi A.T., Gborigo J.T., Aluko R.E., Girgih A.T. (2021). Comparative evaluation of the antioxidant properties of whole peanut flour, defatted peanut protein meal, and peanut protein concentrate. Front. Sustain. Food Syst..

[bib12] Assatory A., Vitelli M., Rajabzadeh A.R., Legge R.L. (2019). Dry fractionation methods for plant protein, starch and fiber enrichment: a review. Trends Food Sci. Technol..

[bib13] Badia-Olmos C., Laguna L., Haros C.M., Tárrega A. (2023). Techno-functional and rheological properties of alternative plant-based flours. Foods.

[bib14] Baginsky C., Peña-Neira Á., Cáceres A., Hernández T., Estrella I., Morales H., Pertuzé R. (2013). Phenolic compound composition in immature seeds of fava bean (Vicia faba L.) varieties cultivated in Chile. J. Food Compos. Anal..

[bib15] Barać M., Pešić M., Stanojević S., Kostić A., Čabrilo S.B. (2015). Techno-functional properties of pea (pisum sativum) protein isolates: a review. Acta Period. Technol..

[bib16] Barbana C., Boye J.I. (2013). In vitro protein digestibility and physico-chemical properties of flours and protein concentrates from two varieties of lentil (lens culinaris). Food Funct..

[bib17] Bautista-Expósito S., Vandenberg A., Dueñas M., Peñas E., Frias J., Martínez-Villaluenga C. (2022). Selection of enzymatic treatments for upcycling lentil hulls into ingredients rich in oligosaccharides and free phenolics. Molecules.

[bib18] BeMiller J.N. (2011). Pasting, paste, and gel properties of starch–hydrocolloid combinations. Carbohydr. Polym..

[bib19] Bloot A.P.M., Kalschne D.L., Amaral J.A.S., Baraldi I.J., Canan C. (2023). A review of phytic acid sources, obtention, and applications. Food Rev. Int..

[bib20] Bourré L., Frohlich P., Young G., Borsuk Y., Sopiwnyk E., Sarkar A., Nickerson M.T., Ai Y., Dyck A., Malcolmson L. (2019). Influence of particle size on flour and baking properties of yellow pea, navy bean, and red lentil flours. Cereal Chem..

[bib21] Boye J., Aksay S., Roufik S., Ribéreau S., Mondor M., Farnworth E., Rajamohamed S. (2010). Comparison of the functional properties of pea, chickpea and lentil protein concentrates processed using ultrafiltration and isoelectric precipitation techniques. Food Res. Int..

[bib22] Boye J., Zare F., Pletch A. (2010). Pulse proteins: processing, characterization, functional properties and applications in food and feed. Food Res. Int..

[bib23] Bozkır E., Santamarina C., Mariotti M., Saia S. (2023). Resistant starch in common beans: concentration, characteristics, uses and health effects. A systematic map and review of the studies from 1962 to 2023. Int. J. Food Sci. Technol..

[bib24] Bubelova Z., Sumczynski D., Salek R.N. (2018). Effect of cooking and germination on antioxidant activity, total polyphenols and flavonoids, fiber content, and digestibility of lentils (lens culinaris L.). J. Food Process. Preserv..

[bib25] Bühler J.M., Dekkers B.L., Bruins M.E., Van Der Goot A.J. (2020). Modifying faba bean protein concentrate using dry heat to increase water holding capacity. Foods.

[bib26] Buscajoni L., Martinetz M.C., Berkemeyer M., Brocard C. (2022). Refolding in the modern biopharmaceutical industry. Biotechnol. Adv..

[bib27] Chang L., Gu Z., Bandillo N., Chen B., Rao J. (2023). Fractionation, structural characteristics, functionality, aromatic profile, and in vitro digestibility of lentil (lens culinaris) proteins. ACS Food Sci. Technol..

[bib28] Chasquibol N., Sotelo A., Tapia M., Alarcon R., Goycoolea F., Hernández-Álvarez A. (2025). Evaluation of cushuro (Nostoc sphaericum) as an alternative source of minerals, functional protein and bioactive peptides. LWT.

[bib29] Costantini M., Sabovics M., Galoburda R., Kince T., Straumite E., Summo C., Pasqualone A. (2021). Effect of die configuration on the physico-chemical properties, anti-nutritional compounds, and sensory features of legume-based extruded snacks. Foods.

[bib30] Crowley S.V., Desautel B., Gazi I., Kelly A.L., Huppertz T., O'Mahony J.A. (2015). Rehydration characteristics of milk protein concentrate powders. J. Food Eng..

[bib31] De Angelis D., Latrofa V., Squeo G., Pasqualone A., Summo C. (2024). Techno-functional, rheological, and chemical properties of plant-based protein ingredients obtained with dry fractionation and wet extraction. Curr. Res. Food Sci..

[bib32] De Angelis D., Pasqualone A., Allegretta I., Porfido C., Terzano R., Squeo G., Summo C. (2021). Antinutritional factors, mineral composition and functional properties of dry fractionated flours as influenced by the type of pulse. Heliyon.

[bib34] De Angelis D., Pasqualone A., Manfredi L., Allegretta I., Terzano R., Summo C. (2022). Dry fractionation as a promising technology to reuse the physically defected legume-based gluten-free pasta. Int. J. Food Sci. Technol..

[bib35] De Mejia E.G., Valadez-Vega M.D.C., Reynoso-Camacho R., Loarca-Pina G. (2005). Tannins, trypsin inhibitors and lectin cytotoxicity in tepary (Phaseolus acutifolius) and common (Phaseolus vulgaris) beans. Plant Foods Hum. Nutr..

[bib36] de Paiva Gouvêa L., Caldeira R., de Lima Azevedo T., Galdeano M.C., Felberg I., Lima J.R., Mellinger C.G. (2023). Physical and techno-functional properties of a common bean protein concentrate compared to commercial legume ingredients for the plant-based market. Food Hydrocoll..

[bib37] de Paiva Gouvêa L., Caldeira R.F., de Lima Azevedo T., Antoniassi R., Galdeano M.C., Felberg I., Lima J.R., Mellinger C.G. (2024). Nutritional properties of common bean protein concentrate compared to commercial legume ingredients for the plant-based market. Curr. Res. Food Sci..

[bib38] Deglaire A., Moughan P.J. (2012). Animal models for determining amino acid digestibility in Humans–A review. Br. J. Nutr..

[bib39] Dida Bulbula D., Urga K. (2018). Cogent food & agriculture. Study on the effect of traditional processing methods on nutritional composition and anti nutritional factors in chickpea (Cicer arietinum).

[bib40] do Carmo C.S., Silventoinen P., Nordgård C.T., Poudroux C., Dessev T., Zobel H., Holtekjølen A.K., Draget K.I., Holopainen-Mantila U., Knutsen S.H. (2020). Is dehulling of peas and faba beans necessary prior to dry fractionation for the production of protein-and starch-rich fractions? Impact on physical properties, chemical composition and techno-functional properties. J. Food Eng..

[bib41] Dong W., Zhang X., Ding L., Liu C., Ai M., Jin Y., Isobe K., Handa A., Cai Z. (2023). Enhancement of emulsification properties by modulation of egg white protein fibril structure with different heating times. Food Hydrocoll..

[bib189] Du S., Jiang H., Yu X., Jane J. (2014). Physicochemical and functional properties of whole legume flour. LWT-Food Sci. Technol..

[bib42] Dumoulin L., Jacquet N., Malumba P., Richel A., Blecker C. (2021). Dry and wet fractionation of plant proteins: how a hybrid process increases yield and impacts nutritional value of faba beans proteins. Innov. Food Sci. Emerg. Technol..

[bib43] Duranti M. (2006). Grain legume proteins and nutraceutical properties. Fitoterapia.

[bib44] Erbersdobler H., Barth C., Jahreis G. (2017). Legumes in human nutrition. Nutrient content and protein quality of pulses. Ernahrungs Umsch..

[bib45] FAO/WHO (1991).

[bib46] Feizollahi E., Mirmahdi R.S., Zoghi A., Zijlstra R.T., Roopesh M., Vasanthan T. (2021). Review of the beneficial and anti-nutritional qualities of phytic acid, and procedures for removing it from food products. Food Res. Int..

[bib47] Fenn D., Wang N., Maximiuk L. (2022). Physicochemical, anti‐nutritional, and functional properties of air‐classified protein concentrates from commercially grown Canadian yellow pea (pisum sativum) varieties with variable protein levels. Cereal Chem..

[bib48] Fenwick D.E., Oakenfull D. (1983). Saponin content of food plants and some prepared foods. J. Sci. Food Agric..

[bib49] Fitzkee N.C., Rose G.D. (2004). Reassessing random-coil statistics in unfolded proteins. Proc. Natl. Acad. Sci..

[bib50] García-Alonso A., Goni I., Saura-Calixto F. (1998). Resistant starch and potential glycaemic index of raw and cooked legumes (lentils, chickpeas and beans). Z. Lebensm. Unters. Forsch. A.

[bib51] Gemede H.F., Ratta N. (2018). Anti dietary factors in plant foods: potential health benefits and adverse effects. Advanced Research Journal of Microbiology.

[bib52] Glusac J., Isaschar-Ovdat S., Fishman A. (2020). Transglutaminase modifies the physical stability and digestibility of chickpea protein-stabilized oil-in-water emulsions. Food Chem..

[bib53] Goertzen A.D., House J.D., Nickerson M.T., Tanaka T. (2021). The impact of enzymatic hydrolysis using three enzymes on the nutritional properties of a chickpea protein isolate. Cereal Chem..

[bib54] Goldstein N., Reifen R. (2022). The potential of legume-derived proteins in the food industry. Grain & Oil Science and Technology.

[bib55] Guo Y., Ma C., Xu Y., Du L., Yang X. (2024). Food gels based on polysaccharide and protein: preparation, formation mechanisms, and delivery of bioactive substances. Gels.

[bib56] Hall A.E., Moraru C.I. (2021). Structure and function of pea, lentil and faba bean proteins treated by high pressure processing and heat treatment. LWT.

[bib57] Hande P.A., Mondal S., Badigannavar A., D'Souza S. (2013). Genetic variability of phytic acid phosphorus and inorganic phosphorus in cultivated groundnut (Arachis hypogaea L.). Plant Genetic Resources.

[bib58] Hendriks W. (2018). Amino acid availability in heat-damaged ingredients. J. Anim. Sci..

[bib59] Herreman L., Nommensen P., Pennings B., Laus M.C. (2020). Comprehensive overview of the quality of plant‐And animal‐sourced proteins based on the digestible indispensable amino acid score. Food Sci. Nutr..

[bib60] Hertzler S.R., Lieblein-Boff J.C., Weiler M., Allgeier C. (2020). Plant proteins: assessing their nutritional quality and effects on health and physical function. Nutrients.

[bib61] Ho T.M., Zhu J., Bansal N., Boyce M.C., Le T.T. (2021). Effect of pH and heat treatment on physicochemical and functional properties of spray-dried whey protein concentrate powder. Int. Dairy J..

[bib62] Hoffman J.R., Falvo M.J. (2004). Protein–which is best?. J. Sports Sci. Med..

[bib63] Hopf A., Agarwal D., Skylas D.J., Whiteway C., Buckow R., Dehghani F. (2024). Techno‐functional properties of dry and wet fractionated pulse protein ingredients. Legume Science.

[bib64] Hu J., Yang J., Xu Y., Zhang K., Nishinari K., Phillips G.O., Fang Y. (2019). Comparative study on foaming and emulsifying properties of different beta-lactoglobulin aggregates. Food Funct..

[bib65] Iqbal A., Khalil I.A., Ateeq N., Khan M.S. (2006). Nutritional quality of important food legumes. Food Chem..

[bib66] Jagannadham K., Parimalavalli R., Babu A.S., Rao J.S. (2014). A study on comparison between cereal (wheat) and non cereal (Chickpea) flour characteristics. International Journal Current Trend Research.

[bib67] Jeganathan B., Vasanthan T., Temelli F. (2024). Mild extraction of faba bean (Vicia faba L.) proteins against conventional methods: impact on physicochemical and thermal characteristics. Food Chem..

[bib68] Jiang Z.-q., Pulkkinen M., Wang Y.-j., Lampi A.-M., Stoddard F.L., Salovaara H., Piironen V., Sontag-Strohm T. (2016). Faba bean flavour and technological property improvement by thermal pre-treatments. LWT--Food Sci. Technol..

[bib69] Jo Y.-J., Huang W., Chen L. (2020). Fabrication and characterization of lentil protein gels from fibrillar aggregates and the gelling mechanism study. Food Funct..

[bib70] Kamani M.H., Liu J., Fitzsimons S.M., Fenelon M.A., Murphy E.G. (2024). Determining the influence of fava bean pre-processing on extractability and functional quality of protein isolates. Food Chem. X.

[bib71] Karaca A.C., Low N., Nickerson M. (2011). Emulsifying properties of chickpea, faba bean, lentil and pea proteins produced by isoelectric precipitation and salt extraction. Food Res. Int..

[bib72] Kaur M., Sandhu K.S. (2010). Functional, thermal and pasting characteristics of flours from different lentil (lens culinaris) cultivars. J. Food Sci. Technol..

[bib73] Kaur M., Singh N. (2005). Studies on functional, thermal and pasting properties of flours from different chickpea (Cicer arietinum L.) cultivars. Food Chem..

[bib74] Kaur M., Singh N. (2007). Characterization of protein isolates from different Indian chickpea (Cicer arietinum L.) cultivars. Food Chem..

[bib75] Khattab R., Arntfield S. (2009). Functional properties of raw and processed canola meal. LWT--Food Sci. Technol..

[bib76] Kottage S.M., Samaranayaka A.G.P., Bhowmik P., Chen L. (2024). Comparison of dry (air classification) and wet fractionated pea protein on protein molecular structure and gelling properties. Sustainable Food Proteins.

[bib77] Labba I.-C.M., Frøkiær H., Sandberg A.-S. (2021). Nutritional and antinutritional composition of fava bean (Vicia faba L., Var. minor) cultivars. Food Res. Int..

[bib78] Laemmli U.K. (1970). Cleavage of structural proteins during the assembly of the head of bacteriophage T4. Nature.

[bib79] Lazarte C.E., Carlsson N.-G., Almgren A., Sandberg A.-S., Granfeldt Y. (2015). Phytate, zinc, iron and calcium content of common Bolivian food, and implications for mineral bioavailability. J. Food Compos. Anal..

[bib80] Lee H.W., Lu Y., Zhang Y., Fu C., Huang D. (2021). Physicochemical and functional properties of red lentil protein isolates from three origins at different pH. Food Chem..

[bib81] Li Z., Messina V., Skylas D.J., Valtchev P., Whiteway C., Cheng S., Langrish T.A., Quail K.J., Dehghani F. (2024). Effect of dry and wet fractionation on nutritional and physicochemical properties of faba bean and yellow pea protein. Legume Science.

[bib82] Liu K., Seegers S., Cao W., Wanasundara J., Chen J., da Silva A.E., Ross K., Franco A.L., Vrijenhoek T., Bhowmik P. (2021). An international collaborative study on trypsin inhibitor assay for legumes, cereals, and related products. J. Am. Oil Chem. Soc..

[bib83] Liu R., Flanagan B.M., Ratanpaul V., Gidley M.J. (2025). Valorising legume protein extraction side-streams: isolation and characterisation of fibre-rich and starch-rich co-products from wet fractionation of five legumes. Food Hydrocoll..

[bib84] Liu S., Ren Y., Yin H., Nickerson M., Pickard M., Ai Y. (2022). Improvement of the nutritional quality of lentil flours by infrared heating of seeds varying in size. Food Chem..

[bib85] Liu Y., Wu X., Hou W., Li P., Sha W., Tian Y. (2017). Structure and function of seed storage proteins in faba bean (Vicia faba L.). 3 Biotech.

[bib86] Ma K.K., Greis M., Lu J., Nolden A.A., McClements D.J., Kinchla A.J. (2022). Functional performance of plant proteins. Foods.

[bib87] Ma Z., Mondor M., Goycoolea F.M., Ganji S.R., Hernández-Álvarez A.J. (2024). Unlocking the potential of waxworm (Galleria mellonella) proteins: extraction, fractionation, and protein quality assessment. Food Biosci..

[bib88] Makri E., Papalamprou E., Doxastakis G. (2005). Study of functional properties of seed storage proteins from Indigenous European legume crops (Lupin, pea, broad bean) in admixture with polysaccharides. Food Hydrocoll..

[bib89] Manzanilla-Valdez M.L., Boesch C., Martinez-Villaluenga C., Montaño S., Hernández-Álvarez A.J. (2024). Enhancing quinoa (Chenopodium quinoa willd) protein extraction: alkaline solubilization coupled to isoelectric precipitation effects on structure, digestibility and antinutrients. Food Hydrocolloids for Health.

[bib90] Manzanilla-Valdez M.L., Boesch C., Orfila C., Montaño S., Hernández-Álvarez A.-J. (2024). Unveiling the nutritional spectrum: a comprehensive analysis of protein quality and antinutritional factors in three varieties of quinoa (Chenopodium quinoa wild). Food Chem. X.

[bib91] Manzanilla-Valdez M.L., Ma Z., Mondor M., Hernández-Álvarez A.J. (2024). Decoding the duality of antinutrients: assessing the impact of protein extraction methods on plant-based protein sources. J. Agric. Food Chem..

[bib93] Marchini M., Carini E., Cataldi N., Boukid F., Blandino M., Ganino T., Vittadini E., Pellegrini N. (2021). The use of red lentil flour in bakery products: how do particle size and substitution level affect rheological properties of wheat bread dough?. LWT.

[bib94] Millar K.A., Gallagher E., Burke R., McCarthy S., Barry-Ryan C. (2019). Proximate composition and anti-nutritional factors of fava-bean (vicia faba), green-pea and yellow-pea (Pisum sativum) flour. J. Food Compos. Anal..

[bib95] Mir N.A., Riar C.S., Singh S. (2019). Effect of pH and holding time on the characteristics of protein isolates from chenopodium seeds and study of their amino acid profile and scoring. Food Chem..

[bib96] Moll P., Salminen H., Griesshaber E., Schmitt C., Weiss J. (2022). Homogenization improves foaming properties of insoluble pea proteins. J. Food Sci..

[bib97] Möller A.C., van der Padt A., van der Goot A.J. (2021). From raw material to mildly refined ingredient–linking structure to composition to understand fractionation processes. J. Food Eng..

[bib98] Möller A.C., van der Padt A., van der Goot A.J. (2022). Influence of the fractionation method on the protein composition and functional properties. Innov. Food Sci. Emerg. Technol..

[bib99] Mondor M., Aksay S., Drolet H., Roufik S., Farnworth E., Boye J.I. (2009). Influence of processing on composition and antinutritional factors of chickpea protein concentrates produced by isoelectric precipitation and ultrafiltration. Innov. Food Sci. Emerg. Technol..

[bib100] Mondor M., Hernández-Álvarez A.J. (2022). Plant Protein Foods.

[bib101] Nasrollahzadeh F., Roman L., Swaraj V.S., Ragavan K., Vidal N.P., Dutcher J.R., Martinez M.M. (2022). Hemp (Cannabis sativa L.) protein concentrates from wet and dry industrial fractionation: molecular properties, nutritional composition, and anisotropic structuring. Food Hydrocoll..

[bib102] Nosworthy M.G., Franczyk A., Neufeld J., House J.D. (2023). The in vivo and in vitro protein quality of three hemp protein sources. Food Sci. Nutr..

[bib103] Nosworthy M.G., Hernandez‐Alvarez A.J., Franczyk A.J., Medina G., Neufeld J., Arcand Y., Ribéreau S., Sánchez‐Velázquez O.A., House J.D. (2023). Effect of cooking on the in vitro and in vivo protein quality of soy, oat and wheat varieties. Cereal Chem..

[bib104] Nosworthy M.G., Medina G., Franczyk A.J., Neufeld J., Appah P., Utioh A., Frohlich P., House J.D. (2018). Effect of processing on the in vitro and in vivo protein quality of red and green lentils (lens culinaris). Food Chem..

[bib105] O'Donoghue L.T., Haque M.K., Kennedy D., Laffir F.R., Hogan S.A., O'Mahony J.A., Murphy E.G. (2019). Influence of particle size on the physicochemical properties and stickiness of dairy powders. Int. Dairy J..

[bib106] Olakanmi S.J., Jayas D.S., Paliwal J., Aluko R.E. (2024). Impact of particle size on the physicochemical, functional, and in vitro digestibility properties of fava bean flour and bread. Foods.

[bib107] Oluwajuyitan T.D., Aluko R.E. (2024). Effect of protein content and particle size on functional properties of air-classified fava bean flour fractions. Food Bioprocess Technol..

[bib108] Oser B.L. (1959). An integrated essential amino acid index for predicting the biological value of proteins. Protein and amino acid nutrition.

[bib109] Ozolina K., Sarenkova I., Muizniece-Brasava S. (2024). Estimation of roasted and raw faba bean and lentil flour functional properties. Food Nutr. J.

[bib110] Pelgrom P.J., Berghout J.A., van der Goot A.J., Boom R.M., Schutyser M.A. (2014). Preparation of functional lupine protein fractions by dry separation. LWT--Food Sci. Technol..

[bib111] Pelgrom P.J., Boom R.M., Schutyser M.A. (2015). Functional analysis of mildly refined fractions from yellow pea. Food Hydrocoll..

[bib112] Pelgrom P.J., Boom R.M., Schutyser M.A. (2015). Method development to increase protein enrichment during dry fractionation of starch-rich legumes. Food Bioprocess Technol..

[bib113] Pelgrom P.J., Vissers A.M., Boom R.M., Schutyser M.A. (2013). Dry fractionation for production of functional pea protein concentrates. Food Res. Int..

[bib114] Pernollet J.-C. (1978). Protein bodies of seeds: ultrastructure, biochemistry, biosynthesis and degradation. Phytochemistry.

[bib115] Pico J., Pismag R.Y., Laudouze M., Martinez M.M. (2020). Systematic evaluation of the folin–ciocalteu and fast blue BB reactions during the analysis of total phenolics in legumes, nuts and plant seeds. Food Funct..

[bib116] Portari G.V., Tavano O.L., Silva M.A.d., Neves V.A. (2005). Effect of chickpea (Cicer arietinum L.) germination on the major globulin content and in vitro digestibility. Food Sci. Technol..

[bib117] Prajapati U., Ksh V., Kumar M., Joshi A. (2021). Handbook of Cereals, Pulses, Roots, and Tubers.

[bib118] Pulivarthi M.K., Buenavista R.M., Bangar S.P., Li Y., Pordesimo L.O., Bean S.R., Siliveru K. (2023). Dry fractionation process operations in the production of protein concentrates: a review. Compr. Rev. Food Sci. Food Saf..

[bib119] Qayyum M., Butt M., Anjum F., Nawaz H. (2012). Composition analysis of some selected legumes for protein isolates recovery. J. Animal & Plant Sci..

[bib120] Qureshi A. (2023). Red Lentil and Green Lentil Flours.

[bib121] Rahma E. (1988). Functional and electrophoretic characteristics of faba bean (Vicia faba) flour proteins as affected by germination. Food Nahrung.

[bib122] Raikos V., Neacsu M., Russell W., Duthie G. (2014). Comparative study of the functional properties of lupin, green pea, fava bean, hemp, and buckwheat flours as affected by pH. Food Sci. Nutr..

[bib123] Raschke T.M. (2006). Water structure and interactions with protein surfaces. Curr. Opin. Struct. Biol..

[bib124] Rempel C., Geng X., Zhang Y. (2019). Industrial scale preparation of pea flour fractions with enhanced nutritive composition by dry fractionation. Food Chem..

[bib125] Rincón F., Martínez B., Ibáñez M.V. (1998). Proximate composition and antinutritive substances in chickpea (Cicer arietinum L) as affected by the biotype factor. J. Sci. Food Agric..

[bib126] Ruckmangathan S., Ganapathyswamy H., Sundararajan A., Thiyagamoorthy U., Green R., Subramani T. (2022). Physico‐chemical, structural, and functional properties of protein concentrate from selected pulses: a comparative study. J. Food Process. Preserv..

[bib127] Ruiz-Ruiz J.C., Dávila-Ortíz G., Chel-Guerrero L.A., Betancur-Ancona D.A. (2012). Wet fractionation of hard-to-cook bean (Phaseolus vulgaris L.) seeds and characterization of protein, starch and fibre fractions. Food Bioprocess Technol..

[bib128] Rullier B., Novales B., Axelos M.A. (2008). Effect of protein aggregates on foaming properties of β-lactoglobulin. Colloids Surf. A Physicochem. Eng. Asp..

[bib129] Saleh H.M., Hassan A.A., Mansour E.H., Fahmy H.A., El-Bedawey A.E.-F.A. (2019). Melatonin, phenolics content and antioxidant activity of germinated selected legumes and their fractions. Journal of the Saudi Society of Agricultural Sciences.

[bib130] Samtiya M., Aluko R.E., Dhewa T. (2020). Plant food anti-nutritional factors and their reduction strategies: an overview. Food Production, Processing and Nutrition.

[bib131] Sánchez-Vioque R., Clemente A., Vioque J., Bautista J., Millán F. (1999). Protein isolates from chickpea (Cicer arietinum L.): Chemical composition, functional properties and protein characterization. Food Chem..

[bib132] Sánchez‐Velázquez O.A., Ribéreau S., Mondor M., Cuevas‐Rodríguez E.O., Arcand Y., Hernández‐Álvarez A.J. (2021). Impact of processing on the in vitro protein quality, bioactive compounds, and antioxidant potential of 10 selected pulses. Legume Science.

[bib133] Schlangen M., Dinani S.T., Schutyser M.A., van der Goot A.J. (2022). Dry fractionation to produce functional fractions from mung bean, yellow pea and cowpea flour. Innov. Food Sci. Emerg. Technol..

[bib134] Schmiele M., Sampaio U.M., Clerici M.T.P.S. (2019). Starches for Food Application.

[bib187] Schutyser M., Novoa S.C., Wetterauw K., Politiek R., Wilms P. (2025). Dry Fractionation for Sustainable Production of Functional, Nutritional and Palatable Grain Legume Protein Ingredients. Food Eng. Rev..

[bib135] Scilingo A.A., Ortiz S.E.M., Martínez E.N., Añón M.a.C. (2002). Amaranth protein isolates modified by hydrolytic and thermal treatments. Relationship between structure and solubility. Food Res. Int..

[bib136] Scott G., Awika J.M. (2023). Effect of protein–starch interactions on starch retrogradation and implications for food product quality. Compr. Rev. Food Sci. Food Saf..

[bib137] Sęczyk Ł., Świeca M., Kapusta I., Gawlik-Dziki U. (2019). Protein–phenolic interactions as a factor affecting the physicochemical properties of white bean proteins. Molecules.

[bib138] Semba R.D., Ramsing R., Rahman N., Kraemer K., Bloem M.W. (2021). Legumes as a sustainable source of protein in human diets. Global Food Secur..

[bib139] Sharma A., Sehgal S. (1992). Effect of processing and cooking on the antinutritional factors of faba bean (Vicia faba). Food Chem..

[bib140] Sharma K., Kaur R., Kumar S., Saini R.K., Sharma S., Pawde S.V., Kumar V. (2023). Saponins: a concise review on food related aspects, applications and health implications. Food Chemistry Advances.

[bib141] Shi D. (2022).

[bib142] Shi D., House J.D., Wanasundara J.P., Nickerson M.T. (2022). Comparative evaluation of the nutritional value of faba bean flours and protein isolates with major legumes in the market. Cereal Chem..

[bib143] Shi D., Nickerson M.T. (2022). Comparative evaluation of the functionality of faba bean protein isolates with major legume proteins in the market. Cereal Chem..

[bib144] Shi L., Arntfield S.D., Nickerson M. (2018). Changes in levels of phytic acid, lectins and oxalates during soaking and cooking of Canadian pulses. Food Res. Int..

[bib145] Silventoinen P., Kortekangas A., Ercili-Cura D., Nordlund E. (2021). Impact of ultra-fine milling and air classification on biochemical and techno-functional characteristics of wheat and rye bran. Food Res. Int..

[bib146] Skylas D.J., Johnson J.B., Kalitsis J., Richard S., Whiteway C., Wesley I., Naiker M., Quail K.J. (2023). Optimised dry processing of protein concentrates from Australian pulses: a comparative study of faba bean, yellow pea and red lentil seed material. Legume Science.

[bib147] Soni K., Samtiya M., Krishnan V., Dhewa T. (2022). Conceptualizing Plant-based Nutrition: Bioresources, Nutrients Repertoire and Bioavailability.

[bib148] Sreerama Y.N., Sashikala V.B., Pratape V.M., Singh V. (2012). Nutrients and antinutrients in cowpea and horse gram flours in comparison to chickpea flour: evaluation of their flour functionality. Food Chem..

[bib149] Srivastava R., Vasishtha H. (2013). Soaking and cooking effect on sapogenols of chickpeas (Cicer arietinum). Current Advances in Agricultural Sciences (An International Journal).

[bib150] Stewart C. (1995). Bubble interaction in low-viscosity liquids. Int. J. Multiphas. Flow.

[bib151] Stone A.K., Karalash A., Tyler R.T., Warkentin T.D., Nickerson M.T. (2015). Functional attributes of pea protein isolates prepared using different extraction methods and cultivars. Food Res. Int..

[bib152] Swanson B.G. (1990). Pea and lentil protein extraction and functionality. JAOCS (J. Am. Oil Chem. Soc.).

[bib153] Tabtabaei S., Jafari M., Rajabzadeh A.R., Legge R.L. (2016). Solvent-free production of protein-enriched fractions from navy bean flour using a triboelectrification-based approach. J. Food Eng..

[bib154] Tang C.-H., Sun X. (2011). A comparative study of physicochemical and conformational properties in three vicilins from phaseolus legumes: implications for the structure–function relationship. Food Hydrocoll..

[bib155] Tang S., Li J., Huang G., Yan L. (2021). Predicting protein surface property with its surface hydrophobicity. Protein Pept. Lett..

[bib156] Tavano O.L., Neves V.A., da Silva Júnior S.I. (2016). In vitro versus in vivo protein digestibility techniques for calculating PDCAAS (protein digestibility-corrected amino acid score) applied to chickpea fractions. Food Res. Int..

[bib157] Thaiphanit S., Schleining G., Anprung P. (2016). Effects of coconut (Cocos nucifera L.) protein hydrolysates obtained from enzymatic hydrolysis on the stability and rheological properties of oil-in-water emulsions. Food Hydrocoll..

[bib158] Thirulogasundar A. (2023). Functional Properties and Protein Quality.

[bib159] Timilsena Y.P., Phosanam A., Stockmann R. (2023). Perspectives on saponins: food functionality and applications. Int. J. Mol. Sci..

[bib160] Tiong A.Y., Crawford S., de Campo L., Ryukhtin V., Garvey C.J., Batchelor W., van’t Hag L. (2025). Legume protein gelation: the mechanism behind the formation of homogeneous and fractal gels. Food Hydrocoll..

[bib161] Tontul I., Topuz A. (2017). Spray-drying of fruit and vegetable juices: effect of drying conditions on the product yield and physical properties. Trends Food Sci. Technol..

[bib162] Trovato M., Funck D., Forlani G., Okumoto S., Amir R. (2021).

[bib163] Vidal-Valverde C., Frias J., Diaz-Pollan C., Fernandez M., Lopez-Jurado M., Urbano G. (1997). Influence of processing on trypsin inhibitor activity of faba beans and its physiological effect. J. Agric. Food Chem..

[bib164] Vogelsang-O’Dwyer M., Petersen I.L., Joehnke M.S., Sørensen J.C., Bez J., Detzel A., Busch M., Krueger M., O'Mahony J.A., Arendt E.K., Zannini E. (2020). Comparison of faba bean protein ingredients produced using dry fractionation and isoelectric precipitation: techno-functional, nutritional and environmental performance. Foods.

[bib165] Wang J., Zheng H., Zhang S., Li J., Zhu X., Jin H., Xu J. (2021). Improvement of protein emulsion stability through glycosylated Black bean protein covalent interaction with (−)-epigallocatechin-3-gallate. RSC Adv..

[bib166] Wang N. (2008). Effect of variety and crude protein content on dehulling quality and on the resulting chemical composition of red lentil (lens culinaris). J. Sci. Food Agric..

[bib167] Wang N., Daun J.K. (2004). Effect of variety and crude protein content on nutrients and certain antinutrients in field peas (pisum sativum). J. Sci. Food Agric..

[bib168] Wang Y., Sánchez-Velázquez O.A., Martínez-Villaluenga C., Goycoolea F.M., Hernández-Álvarez A.J. (2023). Effect of protein extraction and fractionation of chia seeds grown in different locations: nutritional, antinutritional and protein quality assessment. Food Biosci..

[bib169] Wang Y., Sánchez-Velázquez O.A., Martínez-Villaluenga C., Goycoolea F.M., Hernández-Álvarez A.J. (2023). Effect of protein extraction and fractionation of chia seeds grown in different locations: nutritional, antinutritional and protein quality assessment. Food Biosci..

[bib170] Warsame A.O., Michael N., O'Sullivan D.M., Tosi P. (2020). Identification and quantification of major faba bean seed proteins. J. Agric. Food Chem..

[bib171] Withana‐Gamage T.S., Wanasundara J.P., Pietrasik Z., Shand P.J. (2011). Physicochemical, thermal and functional characterisation of protein isolates from kabuli and desi chickpea (Cicer arietinum L.): a comparative study with soy (glycine max) and pea (pisum sativum L.). J. Sci. Food Agric..

[bib172] Wockenfuss L., Lammers V., Heinz V., Sozer N., Silventoinen-Veijalainen P. (2023). Two steps of dry fractionation: Comparison and combination of air classification and electrostatic separation for protein enrichment from defatted rapeseed press cake. J. Food Eng..

[bib173] Xing Q. (2021).

[bib174] Xing Q., Utami D.P., Demattey M.B., Kyriakopoulou K., de Wit M., Boom R.M., Schutyser M.A. (2020). A two-step air classification and electrostatic separation process for protein enrichment of starch-containing legumes. Innov. Food Sci. Emerg. Technol..

[bib175] Xu X., Tao J., Wang Q., Ge J., Li J., Gao F., Gao S., Yang Q., Feng B., Gao J. (2023). A comparative study: functional, thermal and digestive properties of cereal and leguminous proteins in ten crop varieties. LWT.

[bib176] Yadav R.K., Tripathi M.K., Tiwari S. (2024). Estimation of biochemical parameters in chickpea (Cicer arietinum L.) genotypes. Legume Res Int J.

[bib177] Yang J., Yang Q., Waterink B., Venema P., de Vries R., Sagis L.M. (2023). Physical, interfacial and foaming properties of different mung bean protein fractions. Food Hydrocoll..

[bib178] Ye X., Su X., Xiao T., Lu F., Xie T. (2024). High moisture extrusion of soybean protein isolate: effect of β-glucan on physicochemical properties of extrudates. Food Chem..

[bib179] Yust M.a.M., Pedroche J., Girón-Calle J., Vioque J., Millán F., Alaiz M. (2004). Determination of tryptophan by high-performance liquid chromatography of alkaline hydrolysates with spectrophotometric detection. Food Chem..

[bib180] Zehring J., Walter S., Quendt U., Zocher K., Rohn S. (2022). Phytic acid content of faba beans (vicia Faba)—Annual and varietal effects, and influence of organic cultivation practices. Agronomy.

[bib181] Zhang B., Deng Z., Ramdath D.D., Tang Y., Chen P.X., Liu R., Liu Q., Tsao R. (2015). Phenolic profiles of 20 Canadian lentil cultivars and their contribution to antioxidant activity and inhibitory effects on α-glucosidase and pancreatic lipase. Food Chem..

[bib182] Zhang J., Li M., Lv Y., Guo S., Yang B. (2023). Protein aggregation impacts in vitro protein digestibility, peptide profiling and potential bioactive peptides of soymilk and dry-heated soybeans. LWT.

[bib183] Zhang R., Wu W., Zhang Z., Lv S., Xing B., McClements D.J. (2019). Impact of food emulsions on the bioaccessibility of hydrophobic pesticide residues in co-ingested natural products: influence of emulsifier and dietary fiber type. J. Agric. Food Chem..

[bib184] Zhou L., Ali I., Manickam S., Goh B.H., Tao Y., Zhang J., Tang S.Y., Zhang W. (2025). Ultrasound‐induced food protein‐stabilized emulsions: exploring the governing principles from the protein structural perspective. Compr. Rev. Food Sci. Food Saf..

[bib185] Zhu S., Lin S., Ramaswamy H., Yu Y., Zhang Q. (2017). Enhancement of functional properties of rice bran proteins by high pressure treatment and their correlation with surface hydrophobicity. Food Bioprocess Technol..

[bib186] Żmudziński D., Goik U., Ptaszek P. (2021). Functional and rheological properties of Vicia faba L. protein isolates. Biomolecules.

