# Supplementary material for: Industrial-scale fractionation of fava bean, chickpea, and red lentil: A comparative analysis of composition, antinutrients, nutrition, structure, and functionality
Source: Curr Res Food Sci. 2025 Jul 22;11:101152. doi: 10.1016/j.crfs.2025.101152 (PMC12336703; doi:10.1016/j.crfs.2025.101152)
Supplement: Multimedia component 1 [file mmc1.docx]

**Supplementary material for manuscript entitled “Industrial fractionation of legumes: Composition, nutrition, structure, and functionality”**

Ruixian Han, Yan Wang, Zhanming Yang, Stuart Micklethwaite, Martin Mondor, Evi Paximada, Alan Javier Hernández-Álvarez

- 1. **Colour measurement**

Colour of each protein ingredient was measured using a portable colorimeter. The parameters L* indicates lightness, a* indicated red (positive value) and green (negative value), and b* indicated yellow (positive value) and blue (negative value) in the samples. Total colour difference (ΔE^*^) was calculated according to the following equation (Mokrzycki & Tatol, 2011):

ΔE^*^ = $\sqrt{{(L}_{ref}^{*}-L_{sample}^{*})^{2}+{(a}_{ref}^{*}-a_{sample}^{*})^{2}+{(b}_{ref}^{*}-b_{sample}^{*})^{2}}$

Where the raw legume flour without fractionation was used as reference.

***Table S1.*** *Colour (L*, a*, and b*), total colour difference (ΔE^*^) of fava bean, chickpea, and red lentil in three forms: raw flour, dry-fractionation protein-enriched fractions, and wet-fractionated protein-enriched fractions.*

| **Sample** | **Processing** | **L*** | **a*** | **b*** | **ΔE^*^** |
| --- | --- | --- | --- | --- | --- |
| **Fava bean** | **Raw** | 98.56 ± 0.11^b^ | 0.51 ± 0.05^g^ | 9.55 ± 0.06^f^ | - |
|  | **Dry fractionation** | 99.14 ± 0.06^a^ | 1.68 ± 0.13^e^ | 7.66 ± 0.20^g^ | 2.297 |
|  | **Wet fractionation** | 93.65 ± 0.08^f^ | -0.34 ± 0.02^h^ | 15.86 ± 0.10^c^ | 8.040 |
|  |  |  |  |  |  |
| **Chickpea** | **Raw** | 96.09 ± 0.05^e^ | 1.08 ± 0.11^f^ | 14.95 ± 0.14^d^ | - |
|  | **Dry fractionation** | 97.07 ± 0.35^d^ | 0.95 ± 0.05^f^ | 15.63 ± 0.42^cd^ | 1.200 |
|  | **Wet fractionation** | 88.95 ± 0.15^g^ | 4.33 ± 0.08^d^ | 28.33 ± 0.12^a^ | 15.510 |
|  |  |  |  |  |  |
| **Red lentil** | **Raw** | 97.15 ± 0.11^d^ | 12.47 ± 0.33^b^ | 12.03 ± 0.16^e^ | - |
|  | **Dry fractionation** | 97.92 ± 0.18^c^ | 16.36 ± 0.50^a^ | 9.09 ± 0.37^f^ | 4.936 |
|  | **Wet fractionation** | 87.65 ± 0.13^h^ | 11.35 ± 0.07^c^ | 20.44 ± 0.22^b^ | 12.737 |

The reduction in lightness and the shift in colour towards yellowness after wet fraction was obvious. This change was attributed to Maillard reaction under alkaline extraction conditions and high spray-drying temperature (Hopf et al., 2024), as well to the brown quinones formed due to the enhanced extraction of polyphenols (Wintersohle et al., 2023). In contrast, these reactions did not occur during dry fractionation, explaining the minimal changes observed in L* and b* values in dry-fractionated legume PF. Regarding a* value, which is associated with level of natural pigment, including chlorophyll, carotenoids, and anthocyanins (Sousa, 2022), the changes of a* value were largely sample-dependent. Fractionation processing itself did not have a direct effect on the a* value.

- 1. **Pearson correlation coefficient analysis**

***Table S2***. Pearson correlation coefficients (r) among compositional profiles, antinutritional factors, amino acid profiles, protein quality, structural characteristics, protein quality, and techno-functional properties of legumes flours after dry fractionation and wet fractionation.

|  | Dry fractionation | Wet fractionation |
| --- | --- | --- |
| Protein content | 0.9497** | 0.9614** |
| Total starch | -0.9784** | -0.9938*** |
| Available Carbohydrate | -0.8442* | -0.8622* |
| TDF | 0.5111 | -0.9046* |
| Moisture | -0.7450 | -0.8814* |
| TPC | 0.5580 | 0.5761 |
| Phytic acid | 0.5409 | 0.4698 |
| Condensed tannins | 0.9444** | 0.9133* |
| Saponins | 0.8223* | 0.9341** |
| Trypsin inhibitors | 0.7985 | 0.9158* |
| Asp | -0.5621 | -0.7765 |
| Glu | -0.0789 | 0.1051 |
| Ser | 0.1570 | 0.8723* |
| His | 0.2532 | -0.1693 |
| Gly | -0.6330 | -0.8623* |
| Thr | -0.0099 | -0.3921 |
| Arg | 0.5940 | 0.2151 |
| Ala | -0.5448 | -0.5869 |
| Pro | -0.0642 | -0.1627 |
| Tyr | 0.1431 | 0.3542 |
| Val | -0.1605 | -0.4902 |
| Met | 0.4997 | 0.4066 |
| Cys | 0.6923 | 0.5194 |
| IIe | 0.5049 | 0.8695* |
| Trp | -0.8371* | -0.6729 |
| Leu | 0.5838 | 0.9368** |
| Phe | 0.1025 | 0.3799 |
| Lys | 0.3779 | -0.0586 |
| AAS | 0.6699 | 0.4751 |
| EAAI | 0.4660 | 0.1788 |
| BV | 0.4624 | 0.1784 |
| PER_1_ | 0.5609 | 0.9392** |
| PER_2_ | 0.6329 | 0.9497** |
| PER_3_ | 0.9187** | 0.9015* |
| PER_4_ | 0.3181 | 0.2794 |
| PER_5_ | 0.5859 | 0.3907 |
| IVPD | -0.1847 | 0.3716 |
| IVPDCAAS | 0.7175 | 0.6277 |
| D_50_ | -0.8662* | 0.9215** |
| β-sheet | 0.3442 | 0.6112 |
| Random coil | -0.1887 | 0.3243 |
| α-helix | 0.0771 | -0.3379 |
| β-turn | -0.0990 | -0.4935 |
| Surface hydrophobicity | -0.0622 | 0.8973** |
| WHC | 0.5682 | 0.9008** |
| OHC | 0.6388 | -0.3755 |
| FC | -0.1892 | -0.0435 |
| FS | -0.0609 | -0.8653* |
| EAI | 0.6400 | 0.9060** |
| ESI | 0.4424 | 0.0290 |
| LGC | -0.9879*** | -0.8165* |
| Protein solubility (pH7) | -0.9445** | -0.9765** |
| Isoelectric point | 0.3502 | 0.4978 |

*, **, and *** means the difference are significant at the 0.05, 0.01, and 0.001 level, respectively. TDF, total dietary fibre; TPC, total polyphenol content; Gly, glycine; Lys, lysine; Glu, glutamine; Ser, serine; Ala, alanine; Leu, leucine; Met, methionine; Phe, phenylalanine; Trp, tryptophan; Pro, proline; Val, valine; Ile, isoleucine; Cys, cysteine; Tyr, tyrosine; His, histidine; Arg, arginine; Asn, asparagine; Asp, aspartic acid; Thr, threonine; EAAI, essential amino acid index; AAS, amino acid score; BV, predicted biological value; PER_1-5_, Protein efficiency ratio; IVPD, *In vitro* protein digestibility; IVPDCAAS, *In vitro* protein digestibility-corrected amino acid score; D_50_, average particle size; WHC, water holding capacity; OHC, oil holding capacity; FC, foaming capacity; FS, foaming stability; EAI. emulsifying activity index; ESI, emulsifying stability index; PS (pH 7), protein solubility at pH 7; and LGC, least gelation concentration.

**References**

Hopf, A., Agarwal, D., Skylas, D. J., Whiteway, C., Buckow, R., & Dehghani, F. (2024). Techno‐Functional Properties of Dry and Wet Fractionated Pulse Protein Ingredients. *Legume Science, 6*(4), e70005.

Mokrzycki, W., & Tatol, M. (2011). Colour difference∆ EA survey. *Mach. Graph. Vis, 20*(4), 383-411.

Sousa, C. (2022). Anthocyanins, carotenoids and chlorophylls in edible plant leaves unveiled by tandem mass spectrometry. *Foods, 11*(13), 1924.

Wintersohle, C., Kracke, I., Ignatzy, L. M., Etzbach, L., & Schweiggert-Weisz, U. (2023). Physicochemical and chemical properties of mung bean protein isolate affected by the isolation procedure. *Current Research in Food Science, 7*, 100582.
